# An ancestral human genetic variant linked to an ancient disease: A novel association of *FMO2* polymorphisms with tuberculosis (TB) in Ethiopian populations provides new insight into the differential ethno-geographic distribution of FMO2*1

**DOI:** 10.1371/journal.pone.0184931

**Published:** 2017-10-05

**Authors:** Ephrem Mekonnen, Endashaw Bekele

**Affiliations:** 1 Department of Microbial, Cellular, Molecular Biology, Addis Ababa University, Addis Ababa, Ethiopia; 2 Department of Health Biotechnology, Institute of Biotechnology, Addis Ababa University, Addis Ababa, Ethiopia; 3 Department of Microbial, Cellular, Molecular Biology, Addis Ababa University, Addis Ababa, Ethiopia; Cincinnati Children's Hospital Medical Center, UNITED STATES

## Abstract

The human *FMO2* (flavin-containing monooxygenase 2) gene has been shown to be involved in innate immunity against microbial infections, including tuberculosis (TB), via the modulation of oxidative stress levels. It has also been found to possess a curious loss-of-function mutation (FMO2*1/FMO2*2) that demonstrates a distinctive differentiation in expression, function and ethno-geographic distribution. However, despite evidences of ethnic-specific genetic associations in the inflammatory profile of TB, no studies were done to investigate whether these patterns of variations correlate with evidences for the involvement of *FMO2* in antimicrobial immune responses and ethnic differences in the distribution of *FMO2* polymorphisms except for some pharmacogenetic data that suggest a potentially deleterious role for the functional variant (FMO2*1). This genetic epidemiological study was designed to investigate whether there is an association between *FMO2* polymorphisms and TB, an ancient malady that remains a modern global health concern, in a sub-Saharan Africa setting where there is not only a relatively high co-prevalence of the disease and the ancestral FMO2*1 variant but also where both *Mycobcaterium* and *Homo sapiens* are considered to have originated and co-evolved. Blood samples and TB related clinical data were collected from ascertained TB cases and unrelated household controls (n = 292) from 3 different ethnic groups in Ethiopia. Latent *Mtb* infection was determined using Quantiferon to develop reliable TB progression phenotypes. We sequenced exonic regions of *FMO2*.We identified for the first time an association between *FMO2* and TB both at the SNP and haplotype level. Two novel SNPs achieved a study-wide significance [chr1:171181877(A), p = 3.15E-07, OR = 4.644 and chr1:171165749(T), p = 3.32E-06, OR = 6.825] while multiple SNPs (22) showed nominal signals. The pattern of association suggested a protective effect of *FMO2* against both active and latent TB with distinct genetic variants underlying the TB-progression pathway. The results were robust for population stratification. Haplotype-based tests confirmed the SNP-based results with a single haplotype bearing the ancestral-and-functional FMO2*1 "**C**" allele ("AGCTCTACAAT**C**CCCTCGTTGCGC") explaining the overall association (haplotype-specific-p = 0.000103). Strikingly, not only was FMO2*1 nominally associated with reduced risk to "Active TB" (p = 0.0118, OR = 0.496) but it also does not co-segregate with the 5'-3' flanking top high-TB-risk alleles. The study provides an evidence for the existence of an evolutionary adaptation to an ancient disease based on an ancestral genetic variant acting in a haplotypic framework in Ethiopian populations.

## Introduction

### Flavin-containing monooxygenase 2 (*FMO2*)

*FMO2* is a member of a super family of monooxygenase genes. In humans, eleven distinct *FMO* genes exist: five encoding active oxygenases (*FMO1–5*) and six pseudogenes [1]. The former are expressed in a developmental-, sex-, and tissue-specific manner [2]. *FMO2* is the major isoform predominantly and highly expressed in human lung (aka, Pulmonary *FMO*). The lung plays an important role in the metabolism of inhaled foreign chemicals, environmental toxicants, carcinogens, and drugs as well as being the main port of entry, deposition and establishment of inhaled infectious pathogens like *Mtb* [3]. Human *FMO2* possesses an FMO2*1(C)/FMO2*2(T) polymorphism: (g.23238C >T, dbSNP #rs6661174). The ancestral FMO2*1(C) allele encodes for a full-length functionally active enzyme whilst the derived alternate allele, FMO2*2(T), produces a truncated polypeptide that is functionally inactive due to a single-nucleotide transition mutation that converts a glutamine codon to a premature TAG stop codon in exon 9 [4].

### *FMO2* oxygenase activity, oxidative stress and antimycobacterial innate immunity

Several studies have demonstrated the essential role of modulating oxidative stress levels in the innate antimycobacterial immune defense as it affects *Mtb* survival, persistence and subsequent reactivation [5], [6], [7], [8]. Oxygenases in activated macrophages induce oxidative stress through generation of hypoxic conditions and highly reactive oxidants such as reactive oxygen species (ROS). Oxidative stress arises when oxidant load exceeds the endogenous antioxidant capacity. The process occurs in two major stages:1) Oxygenase mediated oxygen uptake leading to oxygen depletion (hypoxia) and, 2) oxygenase mediated generation of oxidizing species leading to the production of cytotoxic free radicals. Hypoxia keeps the aerobic *Mtb* in the latent stage and prevents its proliferation. The free radicals damage almost every part of the target cell (both host and pathogen) through instability and fragmentation of DNAs, proteins and lipids; dysfunction of enzymes; impairment of membrane functions (decreased fluidity, inactivation of membrane-bound receptors, and increased permeability to ions). Although the cytotoxic response of phagocytes causes damage to host tissue (e.g. necrosis), the non-specificity of oxidants is an advantage since it prevents a pathogen from escaping this part of the immune response by mutation of a single molecular target.

In this regard, human *FMO2* (known also as "Pulmonary *FMO*") has been demonstrated to regulate the level of oxidative stress by the generation of metabolites that enhance the release of ROS in the form of H_2_O_2_ [9]. Furthermore, it has been shown that a marked difference exists in ROS leakage from common allelic *FMO2* variants. Another source of evidence for the involvement of *FMO2* in anti-TB immune response through its oxidative potential comes from studies of the role of pharmacogenomics in the treatment of TB.

### FMO2 oxygenase activity, metabolism of antitubercular drugs and pharmacogenomics

Besides pharmacogenomic studies into the general influence of genetic variation in patient response to anti-tubercular drug treatments including the development of serious adverse events [10], several pharmacokinetic studies have focused particularly on the role of *FMO2* [3], [11], [12]. *FMO2* substrates are wide-ranging including therapeutic drugs, dietary-derived compounds and environmental pollutants including thioureas, a widely used class of industrial and pharmaceutical compounds. *FMO2*, through the same basic oxygenase activity that produces immunity-related oxidative stress, metabolizes drug-related exogenous substrates susceptible to oxidation. For example, pharmacogenomic evidences have shed light on how FMO2*1 enzyme functions in relation to the metabolism of the major thiourea-containing anti-MDRTB [defined as TB caused by strains of *Mtb* tuberculosis that are resistant to at least isoniazid and rifampicin [13] drugs such as ethionamide and thiacetazone that result in the production of toxic intermediates [14], [15].

### Ethnic differentiation in TB and FMO2

Several studies in different populations have identified genetic polymorphisms associated with the variable outcome of *Mtb* infection between individuals including African populations [16]. Studies in African populations, particularly sub-Saharan Africa, are important because both *Mtb* and humans are considered to have originated and co-evolved in this sub-continent [17], [18], [19]. Furthermore, studies have demonstrated the existence of ethnic-specific genetic associations with TB [20] as well as differentiation in anti-TB immune response profile between Africans and Europeans [21]. A correspondingly distinctive ethno-geographic differentiation has been shown in the expression and distribution of *FMO2* polymorphisms between African populations and those of non-recent African descent [22], [23], [24]. Particularly, all Europeans and Asians genotyped to date are homozygous for the dysfunctional FMO2*2 allele while, conversely, the functional FMO2*1 variant is found only in Africans (particularly in sub-Saharan Africa), recent African descendants and Hispanics.

In general, the oxygenase activity of the FMO2*1 variant can be described as functioning in an antagonistic pleiotropy vis-à-vis TB: while possessing the *FMO2*.*1* variant helps to fight *Mtb* infection by mounting innate immune responses via the modulation of pulmonary oxidative stress level it also increases the risk of pulmonary toxicity by inducing the adverse metabolism of particular anti-TB drugs. Furthermore, the differential ethno-geographic distribution of FMO2*1 means that there would be a corresponding risk-benefit profile in various populations with regard to resistance to TB and susceptibility to adverse reactions to anti-TB drug treatment. Accordingly, the simultaneous prevalence in sub-Saharan Africa of both high endemic TB and a genetic risk factor for adverse anti-TB drug treatment has led some researchers to characterize FMO2*1 as a "potentially deleterious" variant [22]. In this regard, it was estimated that some 220 million individuals in sub-Saharan Africa may express a functional *FMO2* enzyme and, therefore, potentially at risk of *FMO2* mediated toxicity.

However, to our knowledge, despite evidences suggesting the involvement of *FMO2* in antimicrobial immune response, no studies were done to investigate the "potentially beneficial" aspect of the FMO2*1 variant with regard to TB pathogenesis.

### Rationales and objectives for the study of FMO2 vs. TB

The motivations for the current study are, as outlined above, although expressed *FMO2* has been demonstrated to be involved in the regulation of cellular oxidative stress levels and the generation of ROS that are crucial components of the immune system in the resolution of microbial infections including *Mtb*; although the underlying genetic polymorphisms in *FMO2* responsible for its variable oxygenase activity have been identified; although a distinctive ethno-geographic differentiation in the frequency and expression of *FMO2* polymorphisms have been demonstrated with the ancestral and functional variant, *FMO2*.*1*, exclusively correlating with recent African ancestry reaching its highest frequency in sub-Saharan Africans while Europeans and Asians are homozygous for the derived and non-functional FMO2*2 variant; and, although there are evidences of both ethnic-specific genetic associations and ethnic variation in anti-TB inflammatory response profiles, no studies were done to investigate whether *FMO2* genetic polymorphisms are actually associated with TB susceptibility or resistance patterns.

Therefore, based on these rationales, we decided that FMO2 warrants to be selected as a candidate gene of vital significance in TB pathogenesis and that the results of the study will contribute further to our knowledge of the genetic basis of human variation in TB susceptibility. We hypothesized that *FMO2* polymorphisms may be associated with variation in the outcome of *Mtb* infection and, as a corollary, that this might begin to explain the differential persistence in high proportions of the ancestral FMO2*1 variant responsible for its functionality in sub-Saharan Africa where both *Mtb* and humans are considered to have originated and co-evolved.

## Materials and methods

### Ethical considerations

The research proposal received Ethical Clearance from the relevant institutions in Ethiopia: the Ethical Review Committee of the Department of Biology at Addis Ababa University and the National Health Research Ethics Review Committee of the Federal Ministry of Science and Technology of Ethiopia (Certificate#: RDHE/37-92/2010). Blood samples and other clinical data were collected after obtaining informed and signed consent.

### Study design and setup

A case-control, candidate-gene, household-contact based study was designed and blood samples and TB-related clinical data were collected from unrelated individuals inhabiting three ethno-geographic-categories (EGCs) in Ethiopia: Adigrat (North Ethiopia, Tigrigna speakers, Tigray ethnic group), Merhabete (Central Ethiopia, Amharic speakers, Amhara ethnic group), and Arbaminch (South Ethiopia, Gamingna speakers, Gamo ethnic group). Demographics of the study population is presented in S1 Table. Samples were grouped into four test-model datasets based on strict phenotype definitions:

- test-model 1: "Active TB" (153 cases) vs. "No Active TB" (139 controls)
- test-model 2: "Active TB" (153 cases) vs. "No LTBI" (64 controls)
- test-model 3: "Active TB" (153 cases) vs. "LTBI" (70 controls)
- test-model 4: "LTBI" (70 cases) vs. "No LTBI" (64 controls)

In test-models 1, 2, and 3, cases were patients with 'Active TB' who were hospital-diagnosed (mainly smear-tested pulmonary tuberculosis) and undertaking treatment at the time of sampling (2013–2014). The controls were all unrelated household contacts of patients who have been providing care and/or living in close proximity with the patients in a generally TB endemic setting. Only controls that tested negative for physical symptoms of active pulmonary TB and with no history of TB treatment were included. In test-model 4, cases were individuals with "Latent TB Infection" (LTBI: infection without symptoms of active disease) while controls were individuals with "No Latent TB Infection". 'LTBI' vs. 'No LTBI' status was determined based on *Mtb*-specific whole-blood interferon-gamma release assay (IGRA) (S2 Table). HIV test was performed and all positive samples were excluded. DNA was extracted from buffy-coat and exonic regions of *FMO2* (S13 Table) were sequenced using Illumina Miseq technologies and Homosapiens/UCSC/hg19 release as the reference panel.

### DNA sequence data quality control (QC)

DNA sequence data quality control (QC) and cleaning was performed within the dataset of each test-model and only those markers that passed the QC criteria were included in downstream statistical analyses. QC threshold were selected that maximize individual and marker sizes while ensuring appropriate QC for both. For the per-individual QC, individuals with less than 90% genotyping rate (i.e., individuals missing genotypes for more than 10% of the total markers) were removed. And, as for the per-marker QC, markers with genotyping failure rate of less than 95% (i.e., markers genotyped in less than 95% of all samples) and, markers which fail the Hardy-Weinberg-Equilibrium (HWE) deviation test value of p<0.001 and those with minor allele frequency (MAF) less than 0.01 were all removed. Tests for significant genotyping difference between cases and controls were all negative. These filters resulted in the removal of about 96% of the total nucleotide sequences (7600) mostly because they were monomorphic and thus uninformative for association analysis.

### Statistical tests of association

Various SNP and haplotype based tests of association were performed including covariate and population stratified analyses using PLINK (version 1.07), R (version 3.1.1), and Haploview (version 4.2) software.

1. Basic single SNP association analysis was performed by comparing the minor allele (A1) frequency of individual SNPs between cases and controls. Logistic regression analysis was also done based on additive and genotypic models as well as tests of heterozygote vs. homozygote effects.
2. Covariate analysis was done by including sex, pair-wise EGCs (Merhabete-Adigrat; Merhabete-Arbaminch; Adigrat-Arbaminch) and age in regression models.
3. Test for population specific effects or a difference in the strength of association between EGCs was done as well as tests for allele frequency differences between EGCs.
4. Population stratified analysis was done based on both self-declared ethnicity (EGC) of subjects and homogenous clusters inferred based on identity-by-state (IBS) of SNPs.
5. Conditional haplotype-based association tests were done by specifying a set of SNPs (phenotype-associated) to phase and form haplotypes. Both overall and haplotype-specific association tests were performed.

Generally, the genetic epidemiological analysis was designed to test for both SNP and haplotype based associations with TB progression phenotypes while accounting, or adjusting, for possible confounding factors.

## Results and discussion

The basic tests identified a novel association of multiple *FMO2* SNPs with TB progression phenotypes at varying degrees of significance. The associations identified were suggestive of both TB susceptibility (OR>1: increased risk effect) and resistance (OR<1: decreased risk effect). Only the additive test models were significant and valid. Association test results under the framework of haplotypes also confirmed the robustness of the findings as well as identifying significantly associated susceptibility-/protective-haplotypes. [Note: Detailed data descriptions of sample demographics, IGRA profiles, allele frequency descriptions and results of the various tests of association are presented in the Supporting Information Tables and Figures: S1–S13 Tables and S1–S4 Figs].

### A) Test-models 1, 2, 3: Association test results with "Active TB" as the case-phenotype

A total of twenty two SNPs were significantly associated (p<0.05) with "Active TB" as the case-phenotype. Two SNPs achieved a study-wide significance after Bonferroni correction (P<0.05/94 = 5.32E-4) for multiple comparisons: chr1:171181877(A) [p = 3.15x10^-07^, Bonf.-adjusted-p = 2.96x10^-05^, OR = 4.6, CI = 2.4–8.9] and chr1:171165749(T) [p = 3.32x10^-06^, Bonf.-adjusted p = 3.12x10^-04^, OR = 6.8, CI = 2.6–17.7]. The direction of associations (OR) suggested that there were SNPs with increased (susceptibility) and decreased (resistance) risk to active TB: 8 SNPs with increased risk (Table 1) and 14 SNPs with reduced risk (Table 2). [Note: Functional annotation was done using the UCSC genome browser based on the hg19 reference panel on which the sequencing platform was setup].

**Table 1:** *FMO2* SNPs associated with increased TB risk (Annotation was done using Homosapiens/UCSC/hg19).

**Best results in Active TB vs. No Active TB: SNPs Associated With Susceptibility to Active TB**							
Gene	SNP: Chromosome:Base_position(rsID):Annotation	A1	P	BONF.	OR	L95	U95
*FMO2* (8 SNPs)	chr1:171181877 (Novel variant)	A	3.15E-07	2.96E-05	4.644	2.425	8.893
	chr1:171165749 (Novel variant)	T	3.32E-06	0.000312	6.825	2.63	17.71
	chr1:171179939 (rs3174837)	G	0.02447		2.265	1.111	4.619
	chr1:171180021 (rs6425286)	G	0.02447		2.265	1.111	4.619
	chr1:171174312 (rs16864177)	A	0.03505		1.757	1.035	2.982
	chr1:171178490 (rs28369914)	T	0.03505		1.757	1.035	2.982
	chr1:171168469 (rs112884205)	A	0.04716		3.418	0.9436	12.38
	chr1:171181150 (rs113252377)	A	0.04716		3.418	0.9436	12.38
**Best results in Active TB vs. No LTBI: SNPs Associated With Susceptibility to Active TB**							
Gene	SNP: Chromosome:Base_position(rsID):Annotation	A1	P	BONF.	OR	L95	U95
*FMO2* (2 SNPs)	chr1:171181877 (Novel variant)	A	3.19E-06	0.000261	8.729	2.675	28.48
	chr1:171165749 (Novel variant)	T	6.58E-05	0.005396	15.88	2.149	117.3
**Best results in Active TB vs. LTBI: SNPs Associated With Susceptibility to Active TB**							
Gene	SNP: Chromosome:Base_position(rsID):Annotation	A1	P	BONF.	OR	L95	U95
*FMO2* (4 SNPs)	chr1:171181877 (Novel variant)	A	0.000454	0.04176	5.904	2.188	15.93
	chr1:171165749 (Novel variant)	T	0.000744	0.06848	5.708	1.722	18.92
	chr1:171179939 (rs3174837)	G	0.02233		1.725	1.081	2.754
	chr1:171180021 (rs6425286)	G	0.02233		1.725	1.081	2.754
**Best results in LTBI vs. No LTBI: SNPs Associated With Susceptibility to LTBI**							
Gene	SNP: Chromosome:Base_position(rsID):Annotation	A1	P	BONF.	OR	L95	U95
*FMO2* (2 SNPs)	chr1:171168545 (rs2307492)	C	0.02209		8.514	1.002	72.36
	chr1:171181877 (Novel variant)	A	0.04128		5.41	1.069	27.38

A1: Tested minor allele; P;p-value; Bonf.: Bonferroni-corrected p-value;OR: Odds Ratio; L95/U95: lower/upper 95%CI

**Table 2:** *FMO2* SNPs associated with reduced TB risk (Annotation was done using Homosapiens/UCSC/hg19).

**Best results in Active TB vs. No Active TB: SNPs Associated With Resistance to Active TB**							
Gene	SNP: Chromosome:Base_position(rsID):Annotation	A1	P	BONF.	OR	L95	U95
*FMO2* (12 SNPs)	chr1:171179779 (rs73032526): 3' UTR	G	0.005077		0.5381	0.3488	0.8301
	chr1:171180071 (rs6673781): 3' UTR	G	0.005077		0.5381	0.3488	0.8301
	chr1:171180201 (rs6425286): 3' UTR	C	0.005077		0.5381	0.3488	0.8301
	chr1:171178090 (rs6661174):NONSENSE	C	0.0118		0.3328	0.8768	1
	chr1:171179025 (rs6664553): 3' UTR	C	0.0118		0.3328	0.8768	1
	chr1:171174762 (rs28369899):MISSENSE	C	0.01589		0.1977	0.8646	1
	chr1:171173242 (rs7517460):INTRON	C	0.01722		0.5871	0.3778	0.9124
	chr1:171174691 (rs7536646):SYNONYMOUS	A	0.01882		0.5891	0.3778	0.9185
	chr1:171174821 (rs7536745):INTRON	A	0.01882		0.5891	0.3778	0.9185
	chr1:171176879 (rs6671692):SYNONYMOUS, INTRON	A	0.01882		0.5891	0.3778	0.9185
	chr1:171177858 (rs28369911):INTRON	T	0.02976		0.5167	0.2849	0.9373
	chr1:171179477 (rs7515157):3' UTR	T	0.04895		0.2772	1.001	1
**Best results in Active TB vs. No LTBI: SNPs Associated With Resistance to Active TB**							
Gene	SNP: Chromosome:Base_position(rsID):Annotation	A1	P	BONF.	OR	L95	U95
*FMO2* (5 SNPs)	chr1:171174762 (rs28369899):MISSENSE	C	0.01424		0.1547	0.8398	1
	chr1:171178090 (rs6661174):NONSENSE	C	0.02571		0.496	0.2678	0.9184
	chr1:171179025 (rs6664553):3' UTR	C	0.02571		0.496	0.2678	0.9184
	chr1:171180201 (rs6425286):3' UTR	C	0.0372		0.5516	0.3152	0.9653
	chr1:171179477 (rs7515157):3' UTR	T	0.0404		0.5668	0.3293	0.9755
**Best results in Active TB vs. LTBI: SNPs Associated With Resistance to Active TB**							
Gene	SNP: Chromosome:Base_position(rsID):Annotation	A1	P	BONF.	OR	L95	U95
*FMO2* (5 SNPs)	chr1:171168545 (rs2307492):MISSENSE, INTRON	C	0.02104		0.2088	0.04951	0.8806
	chr1:171179779 (rs73032526):3' UTR	G	0.02401		0.5535	0.3311	0.9251
	chr1:171180071 (rs6673781):3' UTR	G	0.02401		0.5535	0.3311	0.9251
	chr1:171180201 (rs6425286):3' UTR	C	0.03834		0.5836	0.3506	0.9715
	chr1:171154303 (rs28369794):UPSTREAM_VARIANT	C	0.03999		0.1659	0.9732	1
**Best results in LTBI vs. No LTBI: SNPs Associated With Resistance to LTBI**							
Gene	SNP: Chromosome:Base_position(rsID):Annotation	A1	P	BONF.	OR	L95	U95
*FMO2* (5 SNPs)	chr1:171179287 (rs7512785):3' UTR	T	0.02554		0.03839	0.9195	1
	chr1:171179670 (rs28369918):3' UTR	G	0.04233		0.04317	1.054	1
	chr1:171179939 (rs3174837):3' UTR, SPLICE_SITE, INTRON	G	0.04399		0.3466	0.1229	0.9775
	chr1:171180021 (rs6425286):3' UTR	G	0.04399		0.3466	0.1229	0.9775
	chr1:171179477 (rs7515157):3' UTR	T	0.04544		0.1152	0.9999	1

A1: Tested minor allele; P;p-value; Bonf.: Bonferroni-corrected p-value;OR: Odds Ratio; L95/U95: lower/upper 95%CI

### B) Test-model 4: Association test results with "LTBI" as the case-phenotype

A total of seven SNPs were nominally associated to "LTBI" as the case-phenotype with a best result of chr1:171168545(C), p = 2.21E-02, OR = 8.5, CI = 1.0–72.4. Similar to the SNPs linked to active TB, there were SNPs with increased and decreased risk effect to LTBI: two with increased risk and five with reduced risk (Tables 1 and 2).

### Covariate analyses

Overall, sex and age covariates had minimal effect on the test statistics while the inclusion of pair-wise EGC had a relatively more pronounced effect for some SNPs in a test-model specific manner resulting in either the loss of some nominal associations or sensing new signals (S3–S6 Tables). In general, the top-SNPs survived these analyses.

### Accounting for the effect of possible population stratification

Besides the pair-wise EGC covariate analysis, to further adjust for any possible between-population and cryptic within-population differentiation, stratified analyses based on both self-declared ethnicity (EGC) and IBS-based homogenous clusters generated from empiric SNP data were done. Tests for SNP-disease association conditional on the clustering generated by IBS analysis (S1 Fig) was employed both in the combined population and within each EGC using the PLINK option to form clusters each containing at least 1 case and 1 control so that it is informative for association with a threshold of 0.01 (i.e., do not merge individuals differing at p<0.01) [25].

Tests within each EGC replicated the association signals of some SNPs, mostly in the Arbaminch population with the highest samples size. Some SNPs were identified within the second largest sample, Adigrat, while the least number of significant associations were identified in the smallest population, Merhabete (S7 Table). Generally, although there was a difference in the number and identity of SNPs identified by the IBS-based and the EGC-based stratified tests of association, the top SNPs of the study survived both tests. These two tests also sensed new SNP-phenotype association signals not identified by the other tests (S3–S6 Tables).

With regard to the important issue of how efficient were the stratified tests of association at adjusting for any cryptic or known stratification, the "genomic inflation factor" (GIF), which is the ratio of the median of the Chi-square statistic to the expected median value [25], was analyzed. Examination of the changes in GIF showed that, association analysis based on pair-wise IBS clustering did a better job at reducing the GIF value to close to 1 even when compared with the GIF value calculated when self-declared ethnicity was used as a stratification variable (S12 Table). This indicates that, although there may be cryptic population stratification, it has minimal distortive effect on the basic statistical findings of this study particularly with regard to the highly significant SNPs and such concerns can be addressed by employing appropriate adjustments.

To summarize, the advantage of IBS-based association test is that it does not rely on trusting self-declared ethnicity and the only option when there is no available information about ethnicity such as ancestry informative markers. The most important observation from the visual inspection of the MDS plots (S1 Fig) was that the IBS-based clustering did not follow self-declared ethno-geographic lines. Instead, within each cluster, individuals from all three EGC populations overlap and are represented in approximate proportions to their respective sample sizes.

### Analysis of patterns of SNP-phenotype association

Analysis of patterns of SNP-phenotype association revealed a particularly interesting nominal association of the FMO2*1 "C" allele [chr1:171178090 (rs6661174)] with reduced risk to "Active TB" (p = 1.18x10-02, OR = 0.33). FMO2*1 is the ancestral and functionally active variant that was previously demonstrated to be associated with adverse reactions to anti-TB (MDRTB) drug treatment and is non-randomly distributed in global populations found in high frequency in sub-Saharan African populations and descendants from these populations in Hispanics. In statistical terms, this would imply that the alternate allele, FMO2*2 "T", is associated with increased risk to "Active TB" (p = 0.011, OR = 1.851). However, in biological terms, the FMO2*2 "T" is a nonsense mutant allele leading to the production of a truncated and dysfunctional polypeptide. Although FMO2*2 "T" allele is found globally, all Europeans and Asians genotyped to date were homozygous for this allele and, therefore, the current result implies, theoretically, that it would render these populations particularly genetically predisposed to TB; conversely, African populations, especially sub-Saharan African populations possessing high proportions of the FMO2*1 "C" allele, would be expected to be relatively genetically protected from the disease [22].

**Fig 1:**
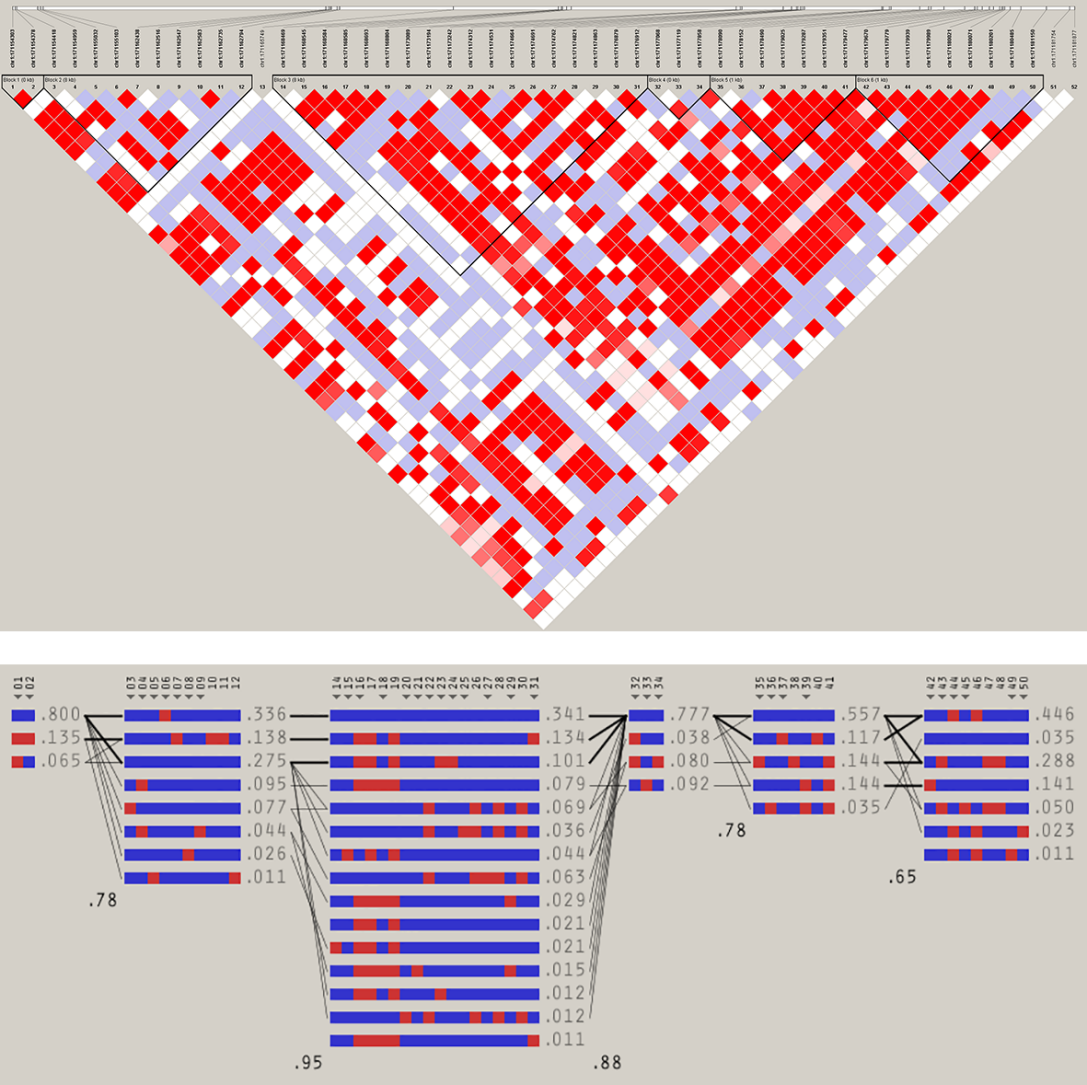
Plots of LD (upper panel) and haplotype block (lower panel) structure of *FMO2* exonic regions based on QC-passed SNP data of the combined population (52 SNPs; 333 samples; genotyping rate of 1).

Another pattern of SNP-phenotype association was that some SNPs exhibit significant association signals in a correlated manner in the sense that two or more SNPs seemed to be associated with the same phenotype concurrently in different test-models. This may indicate that the phenotypes may be influenced by the correlated SNPs acting in concert in a network of biological pathways that lead to the specific associated phenotype, a phenomenon that would be known as epistasis if it occurred at a genic level. On the other hand, the LD structure of the *FMO2* gene may explain correlated association and will be discussed in the next section. In fact, moderate to strong LD (r2 = 0.2–1.0) was observed between some of the phenotype-associated SNPs (Fig 1). [Note: LD-block definition and standard colouring scheme of Haploview were used: Blocks were defined by LD analysis function using the 'Four Gametes Rule'. Colours: white (D' < 1, LOD < 2; recombination); shades of pink/red (D' < 1, LOD > 2; moderate LD); blue (D' = 1, LOD < 2); bright red (D' = 1, LOD > 2; strong LD)].

### Analysis of pair-wise LD patterns between phenotype-associated SNPs

In the presence of strong LD between two or more SNPs, it is logical to expect that these SNPs could produce similar signals of association merely due to their physical proximity or correlated inheritance. Therefore, pair-wise genotypic correlation, r2, was calculated based on genotypic allele counts. With a scale of 0 to 1 (r2 = 0, perfect equilibrium/independence, and r2 = 1 perfect correlation), the two top significantly phenotype-associated SNPs showed not even a moderately strong LD. Rather these SNPs were shown to be located on recombination hotspots that flank regions of high LD and, therefore, it is difficult to attribute a big role to LD for their pattern of consistent association with TB-progression-phenotypes across the test-models. However, it can be seen from the LD plot (Fig 1) and from the almost identical association test statistics generated for some SNPs in LD (S3–S6 Tables), that LD structure had an effect among the other SNPs. The LD and haplotype structure plots in Fig 1 were generated by the "four gametes rule" of Haploview software based on 52 QC-passed SNPs in 333 individuals with a genotyping rate of 1. It is of particular importance to note that the FMO2*1, chr1:171178090(C), allele showed moderate to strong LD (r2 = 0.2–1) with other phenotype-associated SNPs.

### Haplotype-based association analysis

All the statistical tests of association described above were based on individual SNPs. And, although methods based on SNPs lead to significant results, methods based on haplotypes comprising multiple SNPs on the same inherited chromosome may provide additional power for mapping disease loci and also provide insight on factors influencing the dependency among genetic markers [26]. Haplotype-based association can be thought of as a technical validation and refining of a single SNP association signal by framing the test within the haplotypic context of the flanking SNPs, grouping similar haplotypes, and testing for differences in the frequency of the various groups [25]. Therefore, and in light of the LD pattern in *FMO2*, conditional haplotype-based tests were done that involved haplotype inference among the phenotype-associated SNPs discovered by the SNP-based tests using PLINK in all datasets and all haplotypes with > = 0.01 minor-haplotype frequencies. In Table 3, only results showing significant overall (omnibus) and/or haplotype-specific associations are presented. Several haplotypes of the *FMO2* gene were significantly associated with TB-phenotypes: three haplotypes each with 'Active TB' in the 'Active TB vs. No Active TB', in 'Active TB vs. No LTBI', and in 'Active TB vs. LTBI' datasets (the omnibus tests were also significant). Two specific haplotypes were significant in the 'LTBI vs. No LTBI' test-model although the omnibus test was not significant.

**Table 3:** Results of haplotype-based tests of association.

Haplotype-based association test results																											
SNPs																								Haplotype frequency	Test-model	P-value: Haplotype-specific test	P-value: After controlling for specific haplotypes
1	2	3	4	5	6	7	8	9	10	11	12	13	14	15	16	17	18	19	20	21	22	23	24				
A	G	C	T	C	T	A	C	A	A	T	C	C	C	C	T	C	G	T	T	G	C	G	C	0.0453	Active TB vs. No Active TB	0.000103	0.0558
A	**T**	C	T	T	T	G	G	G	G	G	**T**	C	T	C	C	C	A	G	G	A	T	G	**A**	0.0259		0.0127	0.0038
C	G	C	T	T	T	G	G	G	G	G	**T**	C	T	C	C	C	G	T	T	G	C	G	**A**	0.0132		0.0338	0.00216
A	G	C	T	C	T	A	C	A	A	T	C	C	C	C	T	C	G	T	T	G	C	G	C	0.0378	Active TB vs. No LTBI	0.000136	0.0669
A	**T**	C	T	T	A	G	G	G	G	G	**T**	T	T	C	C	C	A	G	G	A	T	G	**A**	0.012		0.0243	0.00388
A	**T**	C	T	T	T	G	G	G	G	G	**T**	C	T	C	C	C	A	G	G	A	T	G	**A**	0.027		0.0381	0.00301
A	G	C	T	C	T	A	C	A	A	T	C	C	C	C	T	C	G	T	T	G	C	G	C	0.0378	Active TB vs. LTBI	0.000136	0.0669
A	**T**	C	T	T	A	G	G	G	G	G	**T**	T	T	C	C	C	A	G	G	A	T	G	**A**	0.012		0.0243	0.00388
A	**T**	C	T	T	T	G	G	G	G	G	**T**	C	T	C	C	C	A	G	G	A	T	G	**A**	0.027		0.0381	0.00301
A	G	C	T	C	T	A	C	A	A	T	C	C	C	C	T	C	G	T	T	G	C	G	C	0.0294	LTBI vs. No LTBI	0.00703	0.442
A	**T**	C	T	T	T	G	G	G	G	G	**T**	C	T	C	C	C	A	G	G	A	T	G	**A**	0.0196		0.0345	0.262

-Within the top row are lists of phenotype-associated SNPs making up each haplotype

-SNP list: SNP1 = chr1:171154303, SNP2 = chr1:171165749, SNP3 = chr1:171168469, SNP4 = chr1:171168545, SNP5 = chr1:171173242, SNP6 = chr1:171174312, SNP7 = chr1:171174691, SNP8 = chr1:171174762, SNP9 = chr1:171174821, SNP10 = chr1:171176879, SNP11 = chr1:171177858, SNP12 = chr1:171178090, SNP13 = chr1:171178490, SNP14 = chr1:171179025, SNP15 = chr1:171179287, SNP16 = chr1:171179477, SNP17 = chr1:171179670, SNP18 = chr1:171179779, SNP19 = chr1:171179939, SNP20 = chr1:171180021, SNP21 = chr1:171180071, SNP22 = chr1:171180201, SNP23 = chr1:171181150, SNP24 = chr1:171181877

-Each row below the SNP list represents a different haplotype; bold = TB-risk alleles

When specific haplotypes were controlled (i.e., effectively left out of the association test model) in order to see if they explain the overall association, one haplotype (AGCTCTACAATCCCCTCGTTGCGC) with a highly significant p value of 0.00703–0.000103, was found to explain the entire association (shown by the rise in its p-value >0.05 while the rest remained significant). This is a remarkable and novel finding in that this particular haplotype contains the well known FMO2*1(**C**) allele.

Moreover, analysis of the inferred haplotypes shows a surprising, but ultimately logical, finding that the FMO2*1 ''C' allele does not appear on the same haplotypic background in combination with its flanking TB-risk alleles. Particularly, the novel and top high-TB-risk alleles, chr1:171181877 (risk allele-"A", protective allele-"C") and chr1:171165749 (risk allele-"T", protective allele-"G") do not co-segregate with the expressed functional ancestral FMO2*1 "C" allele. Instead, these risk alleles segregate with the alternate dysfunctional derived FMO2*2 "T" allele (Table 4). In fact, 67%-75% of the alleles located on the functional "C" haplotype background are associated with TB-resistance. The 'ultimately logical' part of this finding is the fact that in the SNP-based association tests the FMO2*1 "C" allele was found to be negatively associated with Active TB, i.e., it has the effect of reducing risk against "Active TB" phenotype in both test-model 1 and 2 and, hence, the 'GCC' haplotype represents the 'protective haplotype' while the alternative 'TTA' haplotype represents the 'disease haplotype'. This is not surprising from the evolutionary point of view since it is expected that the truncated “T” haplotype will accumulate relatively non-beneficial variants because it is not functionally expressed and therefore there is a relaxation in the selective constraint (no purifying selection). It is also worth restating that Europeans and Asians are homozygous for the FMO2*2 "T" allele. In general, there appears to be a dichotomy of '—C—/—T—' haplotypes associated with decreased and increased risk to Active TB, respectively, although the effect size can be modulated by the proportion of 'risk' vs. 'protective' alleles segregating together on the 'C' haplotype.

**Table 4:** The ancestral and TB-protective FMO2*1 allele does not segregate together with flanking TB susceptibility alleles.

Inferred haplotypes of the *FMO2* gene formed by phenotype-associated SNPs																								
Haplotype	SNPs																							
	1	2	3	4	5	6	7	8	9	10	11	12	13	14	15	16	17	18	19	20	21	22	23	24
**H1**	**A**	**G**	**C**	**T**	**C**	**T**	**A**	**C**	**A**	**A**	**T**	**C**	**C**	**C**	**C**	**T**	**C**	**G**	**T**	**T**	**G**	**C**	**G**	**C**
**H2**	**A**	**G**	**C**	**T**	**C**	**T**	**A**	**G**	**A**	**A**	**G**	**C**	**C**	**C**	**C**	**T**	**C**	**G**	**T**	**T**	**G**	**C**	**G**	**C**
**H3**	**A**	**G**	**C**	**T**	**C**	**T**	**A**	**G**	**A**	**A**	**T**	**C**	**C**	**C**	**C**	**T**	**C**	**G**	**T**	**T**	**G**	**C**	**G**	**C**
**H4**	**A**	**G**	**C**	**T**	**T**	**T**	**G**	**G**	**G**	**G**	**G**	**T**	**C**	**T**	**C**	**C**	**C**	**A**	**G**	**G**	**A**	**T**	**G**	**A**
**H5**	**A**	**G**	**C**	**T**	**T**	**T**	**G**	**G**	**G**	**G**	**G**	**T**	**C**	**T**	**T**	**T**	**G**	**A**	**T**	**T**	**A**	**T**	**G**	**A**
**H6**	**C**	**G**	**C**	**T**	**T**	**T**	**G**	**G**	**G**	**G**	**G**	**T**	**C**	**T**	**C**	**C**	**C**	**G**	**T**	**T**	**G**	**C**	**G**	**A**
**H7**	**A**	**G**	**A**	**T**	**T**	**T**	**G**	**G**	**G**	**G**	**G**	**T**	**C**	**T**	**C**	**C**	**C**	**A**	**G**	**G**	**A**	**T**	**A**	**C**
**H8**	**A**	**G**	**C**	**T**	**C**	**T**	**A**	**G**	**A**	**A**	**G**	**T**	**C**	**T**	**C**	**C**	**C**	**G**	**T**	**T**	**G**	**C**	**G**	**C**
**H9**	**A**	**G**	**C**	**T**	**T**	**A**	**G**	**G**	**G**	**G**	**G**	**T**	**T**	**T**	**C**	**C**	**C**	**A**	**G**	**G**	**A**	**T**	**G**	**C**
**H10**	**A**	**G**	**C**	**T**	**T**	**T**	**G**	**G**	**G**	**G**	**G**	**T**	**C**	**T**	**C**	**C**	**C**	**A**	**G**	**G**	**A**	**T**	**G**	**C**
**H11**	**A**	**G**	**C**	**T**	**T**	**T**	**G**	**G**	**G**	**G**	**G**	**T**	**C**	**T**	**C**	**C**	**C**	**G**	**T**	**T**	**G**	**C**	**G**	**C**
**H12**	**A**	**G**	**C**	**T**	**T**	**T**	**G**	**G**	**G**	**G**	**G**	**T**	**C**	**T**	**T**	**T**	**G**	**A**	**T**	**T**	**A**	**T**	**G**	**C**
**H13**	**C**	**G**	**C**	**C**	**T**	**T**	**G**	**G**	**G**	**G**	**G**	**T**	**C**	**T**	**T**	**T**	**C**	**A**	**T**	**T**	**A**	**T**	**G**	**C**
**H14**	**C**	**G**	**C**	**C**	**T**	**T**	**G**	**G**	**G**	**G**	**G**	**T**	**C**	**T**	**T**	**T**	**G**	**A**	**T**	**T**	**A**	**T**	**G**	**C**
**H15**	**C**	**G**	**C**	**T**	**T**	**A**	**G**	**G**	**G**	**G**	**G**	**T**	**T**	**T**	**C**	**C**	**C**	**A**	**G**	**G**	**A**	**T**	**G**	**C**
**H16**	**C**	**G**	**C**	**T**	**T**	**T**	**G**	**G**	**G**	**G**	**G**	**T**	**C**	**T**	**C**	**C**	**C**	**G**	**T**	**T**	**G**	**C**	**G**	**C**
**H17**	**A**	**T**	**C**	**T**	**T**	**A**	**G**	**G**	**G**	**G**	**G**	**T**	**T**	**T**	**C**	**C**	**C**	**A**	**G**	**G**	**A**	**T**	**G**	**A**
**H18**	**A**	**T**	**C**	**T**	**T**	**T**	**G**	**G**	**G**	**G**	**G**	**T**	**C**	**T**	**C**	**C**	**C**	**A**	**G**	**G**	**A**	**T**	**G**	**A**
**H19**	**C**	**T**	**C**	**T**	**T**	**T**	**G**	**G**	**G**	**G**	**G**	**T**	**C**	**T**	**C**	**C**	**C**	**G**	**T**	**T**	**G**	**C**	**G**	**A**

-Each row below the SNP list row represents an inferred haplotype composed of phenotype-associated SNPs

-red = TB resistant alleles; light green = moderately TB susceptible alleles; dark green = highly TB-risk alleles; blue = SNP chr1:171178090 (rs6661174)

-SNP list: SNP1 = chr1:171154303, SNP2 = chr1:171165749, SNP3 = chr1:171168469, SNP4 = chr1:171168545, SNP5 = chr1:171173242, SNP6 = chr1:171174312, SNP7 = chr1:171174691, SNP8 = chr1:171174762, SNP9 = chr1:171174821, SNP10 = chr1:171176879, SNP11 = chr1:171177858, SNP12 = chr1:171178090 (FMO2*1/FMO2*2), SNP13 = chr1:171178490, SNP14 = chr1:171179025, SNP15 = chr1:171179287, SNP16 = chr1:171179477, SNP17 = chr1:171179670, SNP18 = chr1:171179779, SNP19 = chr1:171179939, SNP20 = chr1:171180021, SNP21 = chr1:171180071, SNP22 = chr1:171180201, SNP23 = chr1:171181150, SNP24 = chr1:171181877

-Each row below the SNP list represents a different haplotype; bold = TB-risk alleles

To summarize, the LD/haplotype-based association tests strongly supported the findings obtained through the basic SNP-based association. This indicates the robustness of the significant associations since, at the very least, the haplotype-based tests preclude possible technical genotyping artefacts that may have influenced the association statistic [25]. Furthermore, it demonstrates how SNPs can act in a haplotypic framework and how haplotypic variation affects complex human disease traits such as TB.

### Allelic and genotypic distribution of FMO2*1/FMO2*2

It has been reported that the presence of other SNPs in *FMO2* gene would not alter FMO2*1's activity in individuals possessing at least one FMO2*1 allele. Therefore, the percentage and distribution of possession of at least one FMO2*1 allele should closely reflect the prevalence of individuals producing active *FMO2* protein [27]. In the present study, the FMO2*2 allele (T) was found to be the major allele while the ancestral FMO2*1 (C) allele remained a minor one in each EGC (S8 Table and S2–S4 Figs). For example, 24% of all individuals genotyped in this study carried at least one of the potentially deleterious FMO2*1 ancestral allele.

### Can the novel finding of an association of FMO2 with TB begin to explain the differential FMO2*1/FMO*2 ethno-geographic distribution?

When one considers the fact that the FMO2*2 mutant, but now major, allele is dysfunctional, and thus selectively neutral, it is tempting to ask if the ancestral, but now minor, allele which codes for a metabolically active protein has a deleterious effect and is thus undergoing a natural purge. For the latter to happen, however, the FMO2*1 allele must be acting to predispose to some highly penetrant, early-onset, and deleterious phenotype. The recent discovery of its association with adverse reactions to industrial and pharmaceutical chemicals alone is insufficient to fully account for its low frequency since these chemicals are relatively recent man-made substrates and have not had much time to apply selective pressure in a differential or population-specific manner. In fact, some studies on the evolution of *FMO*s have also noted the absence of an evidence for such adaptive selection in *FMO2* [28], [29].

In lieu of the purifying selection postulate described above, it is proposed here that one possible explanation for the differential distribution of the *FMO2* alleles is that the FMO2*1 allele need not be considered 'deleterious' (except, of course, vis-a-vis its interaction with some unnatural, recently-manufactured chemicals mentioned above) to explain its low frequency despite being an ancestral allele. To the contrary, as the current study clearly demonstrates, it could have a beneficial protective health effect that could explain its persistence in African populations and their descendants. This proposal is consistent with previous findings [5], [8], [30], [31], [32] that demonstrated the essential role of regulating oxidative stress level in the immune response against mycobacterial infections and studies [9] that showed the involvement of FMO2*1 in regulating biological pathways related to the modulation of cellular oxidative stress status.

In biological and evolutionary terms, the discovery of an ancestral genetic variant that protects against TB is not surprising particularly in TB-endemic populations that were being persistently challenged by an ancient disease with strong selective pressure. Furthermore, it is plausible that, besides the endemicity of TB and the diversity of the causal *Mtb* strains in sub-Saharan Africa, the nature and environmental distribution of other *FMO2* substrates prevalent in this regional settings may act in concert as selective agents that further favour the FMO2*1 structural and functional variant. And, ultimately, as long as it is essential for fitness, once FMO2*1 has been established in particular populations, it will tend to be maintained by natural selection its frequency modulated by its effect size and demographic events. This can result in a pattern of population-specific susceptibility or resistance against TB. This argument is also in keeping with previous findings that reported not only ethnic-specific genetic associations with TB [20] but also variation in the inflammatory response profile among TB patients with different ethnic backgrounds [21]. In this regard, evidences for human adaptation in an ethno-geography specific manner in Ethiopia are not restricted to disease phenotypes. For example, previous studies of Ethiopian populations have demonstrated the existence of an exclusively Ethiopian pattern of genetic adaptation to high-altitude hypoxia [33], [34].

### The double-edged sword of FMO2: Role in TB pathogenesis and anti-TB pharmacogenomics

As has been discussed above, there is evidence that some widely used antitubercular drugs, such as ethionamide and thiacetazone, are substrates of *FMO2*. It has also been reported that the metabolism of ethionamide and thiacetazone by human *FMO2* affects both its efficacy and toxicity [15]. Furthermore, the emergence and spread of MDR and XDR strains of *Mtb* has led to the increased use of such drugs worldwide. For example, two recent studies in Ethiopia [35], [36] found that not only were both MDR- and XDR-TB present in Ethiopian patients but also resistant strains against ethionamide were the most prevalent types.

Therefore, the identification in the present study of *FMO2* SNPs and haplotypes significantly associated with TB disease progression coupled with previous reports of *FMO2*-mediated variation in response to anti-TB drug treatment, calls for more directed studies into the role of *FMO2* in TB pathogenesis and pharmacogenomics. For example, functional annotation of *FMO2* polymorphisms might reveal the immunogenetic basis for its association with both TB disease and treatment outcome. In this regard, a previous study [21] has demonstrated the existence of differential TB immunologic profiles at presentation, becoming even more marked following initiation of antimycobacterial therapy, between patients of African vs. Eurasian ancestry and that associated with ethnic variation in host genotype.

Generally, the current finding demonstrates the intricacies in the spectrum of genetic associations with TB pathogenesis and treatment. From the evolutionary perspective, it is informative to note that how changes in the environment, in this case the manufacturing, distribution and utilization of new drugs to treat an ancient disease, may create a pressure on a genetic architecture evolutionarily shaped to fight the same disease. In other words, it is not a matter of the FMO2*1 allele being naturally deleterious, rather the change in the human environment that is becoming an artificial risk. And how, ultimately, it is the combination of all the factors involved in the resolution of TB infection (some with minor, some with major effects) that determines the outcome. It is the hope of identifying genetic factors with major effects that drives genetic epidemiological investigations like the current study.

## Conclusion

We report the first discovery of an association between *FMO2* genetic polymorphisms and TB progression phenotypes both at the SNP and haplotype levels. We identified multiple SNPs, including novel variants, associated with increased or decreased risk to TB in Ethiopian populations. Furthermore, we found that specific combinations of *FMO2* alleles form either protective or risk haplotypes. A remarkable discovery was that the majority of the alleles associated with TB susceptibility, including the novel and most significantly high-TB-risk alleles, do not co-segregate with the ancestral, expressed and functional FMO2*1 "C" allele which was nominally associated with resistance to TB. Instead, the TB-risk alleles segregate with the derived and dysfunctional FMO2*2 "T" allele and thus are not expressed. On the other hand, 67%-75% of the TB-phenotype associated alleles located on the FMO2*1 "C" haplotype background were associated with TB-resistance and expressed. This pattern of association suggests that the protective effect of FMO2*1 against TB operates in a haplotypic framework with a stronger effect and will enable future experimental validation.

Our novel findings provide an exciting alternative explanation for the present ethno-geographic differentiation of FMO2*1 with high proportions in sub-Saharan Africa, a region where both humans and *Mtb* are considered to have originated and co-evolved. We propose that the study results suggest the existence of an evolutionary adaptation in Ethiopian populations to an ancient disease based on a haplotypic framework involving an ancestral *FMO2* genetic variant.

Our discovery also has an enormous public health implication. Since FMO2*1 mediated toxicity towards some widely used thiourea based anti-TB drugs has previously been demonstrated, we question the prudence of prescribing such treatment regimens for populations harbouring high proportions of FMO2*1 without genetic screening or the development of simpler biomarkers. In this regard, our findings represent a curious paradigm of pleiotropy in action: a locus with genetic polymorphisms that not only protect individuals against a disease but, if a particular class of drugs is administered to treat the disease, it may also lead to adverse reactions. In other words, our findings demonstrate how changes in the environment, in this case the manufacturing, distribution and utilization of new drugs to treat an ancient disease, may create a pressure on a genetic architecture evolutionarily shaped to fight the same disease.

To conclude, the findings of the study provide further insight into the genetic basis of anti-TB immune responses involving the regulation of oxidative stress and calls for a revision of the notion that the FMO2*1 variant is "potentially deleterious". Rather, we propose that the potentially beneficial effect of FMO2*1 against microbial infections may explain its differentially high persistence in sub-Saharan Africa. This conclusion is in line with previous evidences of TB-related differentiation in genetic association signals and immune response profiles between populations of African and non-recent African descent as well as evidences of a correspondingly distinctive ethno-geographic differentiation in the expression and distribution of *FMO2* polymorphisms. Further investigations into the potential existence of genetic signatures of selection for FMO2*1 is essential, particularly in a sub-Saharan setting where both the candidate gene and the disease phenotype are common, to resolve this conclusion. We also recommend that the biological role of polymorphisms in *FMO*s in general, as oxygenases, should be investigated with respect to other disease phenotypes that involve oxidative stress modulation in their pathogenesis. Finally, our study indicates the need for integrating evidences of *Mtb*-human co-evolution in the prevailing hypothesis behind genetic epidemiological investigations of TB that might explain its various signals of ethno-geographic differentiation.

### Limitations of the study

The relatively small samples size of this study is its major limitation which was compounded further by the progressively stricter definitions of case-control phenotypes required for sensitivity analysis. Furthermore, additional samples had to be excluded because they either tested positive for HIV or had indeterminate Quantiferon results, or both, thus reducing power to detect signals of association.

## Supporting information

S1 Fig MDS-Plots of the first two components of multidimensional scaling analysis for the 'Active TB vs. No Active TB' test-model dataset. (DOCX)

S2 Fig Minor allele (FMO2*1) frequency distribution in Ethiopian populations. (DOCX)

S3 Fig Proportions of at least one FMO2*1 allele in Ethiopian populations. (DOCX)

S4 Fig Proportions of FMO2*1/FMO2*2 genotypes in Ethiopian populations. (DOCX)

S1 Table Demographic characteristics of subjects. (DOCX)

S2 Table IGRA results. (DOCX)

S3 Table Association test results in test-model 1. (DOCX)

S4 Table Association test results in test-model 2. (DOCX)

S5 Table Association test results in test-model 3. (DOCX)

S6 Table Association test results in test-model 4. (DOCX)

S7 Table Summary of population-specific SNP-TB phenotype association test results. (DOCX)

S8 Table Allele frequencies of TB phenotype-associated FMO2 SNPs among EGCs. (DOCX)

S9 Table Breslow-Day test results for hetrogeneous associations. (DOCX)

S10 Table Test results for allele frequency differences between EGCs. (DOCX)

S11 Table Functional consequences of mutations risk (Annotation was done using Homosapiens/UCSC/hg19). (DOCX)

S12 Table Comparison of genomic inflation factor. (DOCX)

S13 Table Sequenced regions of FMO2. (DOCX)
