# Supplementary material for: An ancestral human genetic variant linked to an ancient disease: A novel association of FMO2 polymorphisms with tuberculosis (TB) in Ethiopian populations provides new insight into the differential ethno-geographic distribution of FMO2*1
Source: PLoS One. 2017 Oct 5;12(10):e0184931. doi: 10.1371/journal.pone.0184931 (PMC5628799; doi:10.1371/journal.pone.0184931)
Supplement: S1 Fig — (DOCX) [file pone.0184931.s001.docx]

a) b)


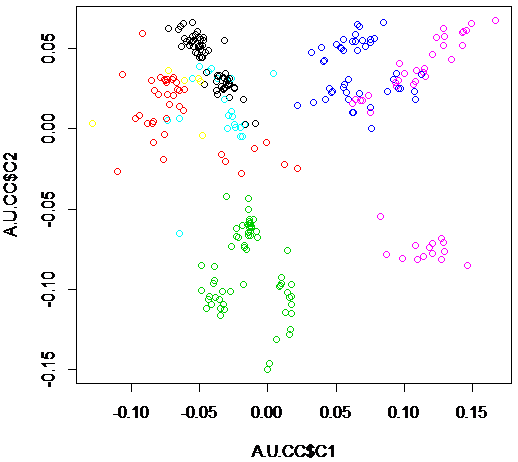

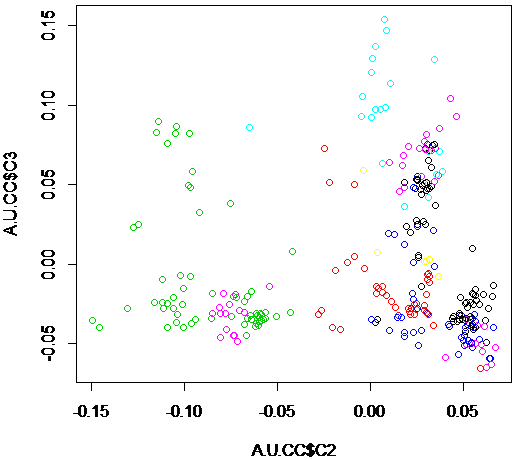


c) d)


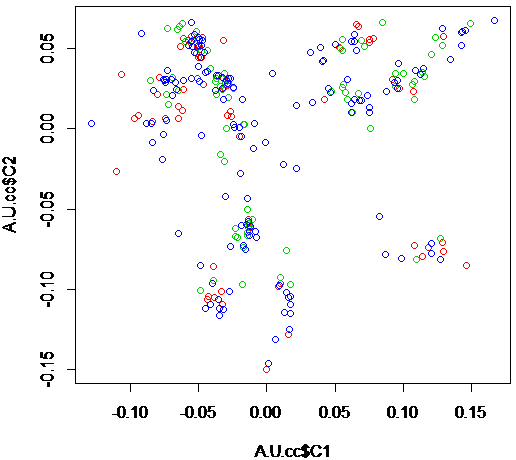

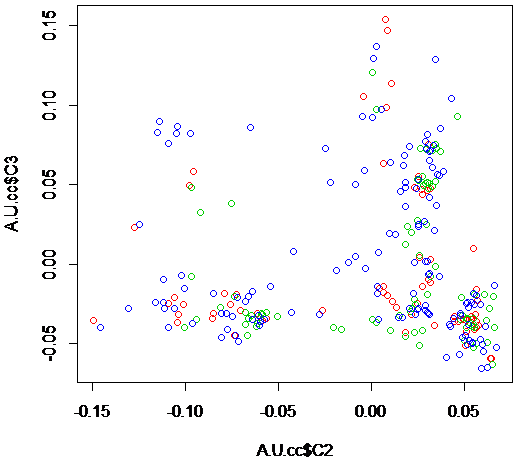


*- A.U.cc= Affected vs. unaffected (case-control); $C1,2,3=MDS components 1,2,3; each circle represents an individual.*

*- a) and b) both clustering analysis and colouring scheme based on empiric genetic data*

*- c) and d) clustering analysis based on empiric genetic data and colouring scheme based on ethnicity (Green=Merhabete; Blue=Adigrat; Red=Arbaminch)*
