# Supplementary material for: An ancestral human genetic variant linked to an ancient disease: A novel association of FMO2 polymorphisms with tuberculosis (TB) in Ethiopian populations provides new insight into the differential ethno-geographic distribution of FMO2*1
Source: PLoS One. 2017 Oct 5;12(10):e0184931. doi: 10.1371/journal.pone.0184931 (PMC5628799; doi:10.1371/journal.pone.0184931)
Supplement: S1 Table — (DOCX) [file pone.0184931.s005.docx]

S Table 1. Demographic characteristics of subjects

| Descriptives | Site (Ethnic group) | Subject status | | | |
| --- | --- | --- | --- | --- | --- |
|  |  | Active TB | No Active TB | QFT positive | QFT negative |
| % Male | Merhabete (Amhara) | 58.1 | 69.2 | 76.2 | 62.5 |
|  | Adigrat (Tigray) | 71.1 | 38.3 | 47.8 | 27.3 |
|  | Arbaminch (Gamo) | 83.3 | 86.8 | 90.0 | 84.4 |
|  | Combined population | 75.2 | 65.5 | 70.3 | 61.4 |
| Mean age (range) | Merhabete (Amhara) | 39.7 (15-70) | 31.0 (22-63) | 30.9 (23-57) | 29.9 (22-55) |
|  | Adigrat (Tigray) | 38.5 (18-85) | 33.2 (15-67) | 40.3 (21-60) | 27.0 (15-67) |
|  | Arbaminch (Gamo) | 30.6 (15-82) | 32.9 (15-105) | 33.3 (15-85) | 32.9 (17-105) |
|  | Combined population | 34.4 (15-85) | 32.5 (15-105) | 35.0 (15-85) | 29.8 (15-105) |
| Sample size (N) | Merhabete (Amhara) | 31 | 39 | 16 | 21 |
|  | Adigrat (Tigray) | 38 | 47 | 22 | 23 |
|  | Arbaminch (Gamo) | 84 | 53 | 32 | 20 |
|  | Combined population | 153 | 139 | 70 | 64 |
