# Supplementary material for: An ancestral human genetic variant linked to an ancient disease: A novel association of FMO2 polymorphisms with tuberculosis (TB) in Ethiopian populations provides new insight into the differential ethno-geographic distribution of FMO2*1
Source: PLoS One. 2017 Oct 5;12(10):e0184931. doi: 10.1371/journal.pone.0184931 (PMC5628799; doi:10.1371/journal.pone.0184931)
Supplement: S2 Table — (DOCX) [file pone.0184931.s006.docx]

S Table 2. IGRA results

| Summary of latent TB infection (LTBI) test result | | | | | | | |
| --- | --- | --- | --- | --- | --- | --- | --- |
| Sampling site | Indeterminate | % | Negative | % | Positive | % | Total |
| Adigrat | 0 | 0 | 26 | 48 | 28 | 52 | 54 |
| Arbaminch | 7 | 11 | 21 | 32 | 38 | 58 | 66 |
| Merhabete | 1 | 2 | 28 | 58 | 19 | 40 | 48 |
| Total | 8 | 5 | 75 | 45 | 85 | 51 | 168 |
