# Supplementary material for: An ancestral human genetic variant linked to an ancient disease: A novel association of FMO2 polymorphisms with tuberculosis (TB) in Ethiopian populations provides new insight into the differential ethno-geographic distribution of FMO2*1
Source: PLoS One. 2017 Oct 5;12(10):e0184931. doi: 10.1371/journal.pone.0184931 (PMC5628799; doi:10.1371/journal.pone.0184931)
Supplement: S3 Table — (DOCX) [file pone.0184931.s007.docx]

S Table 3. Association test results in Test-model 1

| Test-model 1: Active TB vs. No Active TB | | | | | | | | | | | | | | | | | | | | | | | | | | |
| --- | --- | --- | --- | --- | --- | --- | --- | --- | --- | --- | --- | --- | --- | --- | --- | --- | --- | --- | --- | --- | --- | --- | --- | --- | --- | --- |
| Gene | SNP | Minor allele (A1) | Best p | OR | Fisher | | | | Pearson | | | | Logistic regression | | | | Covariate | | | | | Stratified tests (CMH) | | | | |
|  |  |  |  |  | Combined | Merhabete | Adigrat | Arbaminch | Combined | Merhabete | Adigrat | Arbaminch | Combined | Merhabete | Adigrat | Arbaminch | Sex | Age | Mer-Adi | Mer-Arb | Adi-Arb | EGC | IBS | IBS-Mer | IBS-Adi | IBS-Arb |
| FMO2 | chr1:171165749 | T | 3.32E-06 | 6.8 | 3.32E-06 |  |  | 1.90E-04 | 6.72E-06 |  |  | 4.11E-04 | 3.95E-05 |  |  | 5.05E-03 | 4.27E-05 | 3.11E-05 | 1.61E-02 | 1.61E-03 | 5.18E-04 | 1.63E-05 | 8.34E-06 |  |  | 9.46E-04 |
|  | chr1:171168469 | A | 4.72E-02 | 3.4 |  |  |  |  | 4.72E-02 |  |  |  |  |  |  |  |  |  |  |  |  |  |  |  |  |  |
|  | chr1:171174312 | A | 3.51E-02 | 1.8 |  |  |  |  | 4.40E-02 |  |  |  | 4.98E-02 |  |  |  | 4.33E-02 |  |  |  |  | 3.51E-02 |  |  |  |  |
|  | chr1:171178490 | T | 3.51E-02 |  |  |  |  |  | 4.40E-02 |  |  |  | 4.61E-02 |  |  |  | 3.99E-02 | 4.63E-02 |  |  |  | 3.51E-02 |  |  |  |  |
|  | chr1:171179939 | G | 2.45E-02 | 2.3 |  |  | 4.47E-02 |  |  |  | 3.34E-02 |  |  |  | 2.45E-02 |  |  |  |  |  | 2.86E-02 | 3.54E-02 |  |  |  |  |
|  | chr1:171180021 | G | 2.45E-02 | 2.3 |  |  | 4.47E-02 |  |  |  | 3.34E-02 |  |  |  | 2.45E-02 |  |  |  |  |  | 2.86E-02 | 3.54E-02 |  |  |  |  |
|  | chr1:171181150 | A | 4.72E-02 | 3.4 |  |  |  |  | 4.72E-02 |  |  |  |  |  |  |  |  |  |  |  |  |  |  |  |  |  |
|  | chr1:171181877 | A | 3.15E-07 | 4.6 | 3.15E-07 |  |  | 2.12E-06 | 6.03E-07 |  |  | 7.85E-06 | 6.48E-07 |  |  | 1.19E-04 | 7.53E-07 | 4.72E-07 | 2.51E-02 | 1.07E-05 | 3.97E-06 | 1.77E-06 | 2.16E-06 |  |  | 4.12E-05 |
|  | chr1:171173242 | C | *1.72E-02* | *0.59* | *3.71E-02* |  |  |  | *2.97E-02* |  |  |  | *3.60E-02* |  |  |  | *2.59E-02* | *3.84E-02* |  |  | *2.10E-02* | *1.72E-02* |  |  |  |  |
|  | chr1:171174691 | A | *1.88E-02* | *0.59* | *3.56E-02* |  |  |  | *2.92E-02* |  |  |  | *3.63E-02* |  |  |  | *2.71E-02* | *3.83E-02* |  |  | *2.37E-02* | *1.88E-02* |  |  |  |  |
|  | chr1:171174762 | C | *1.59E-02* | *0.20* | *2.05E-02* |  |  |  | *1.59E-02* |  |  |  | *1.93E-02* |  |  |  | *1.87E-02* | *2.08E-02* |  |  | *3.28E-02* |  |  |  |  |  |
|  | chr1:171174821 | A | *1.88E-02* | *0.59* | *3.56E-02* |  |  |  | *2.92E-02* |  |  |  | *3.63E-02* |  |  |  | *2.71E-02* | *3.83E-02* |  |  | *2.37E-02* | *1.88E-02* |  |  |  |  |
|  | chr1:171176879 | A | *1.88E-02* | *0.59* | *3.56E-02* |  |  |  | *2.92E-02* |  |  |  | *3.63E-02* |  |  |  | *2.71E-02* | *3.83E-02* |  |  | *2.37E-02* | *1.88E-02* |  |  |  |  |
|  | chr1:171177858 | T | *2.98E-02* | *0.52* | *3.49E-02* |  |  |  | *3.10E-02* |  |  |  | *4.29E-02* |  |  |  | *2.98E-02* | *4.69E-02* |  |  |  |  |  |  |  |  |
|  | chr1:171178090 | C | *1.18E-02* | *0.33* | *1.51E-02* |  |  |  | *1.18E-02* |  |  |  | *1.70E-02* |  |  |  | *1.39E-02* | *1.74E-02* |  |  | *2.92E-02* | *2.34E-02* |  |  |  |  |
|  | chr1:171179025 | C | *1.18E-02* | *0.33* | *1.51E-02* |  |  |  | *1.18E-02* |  |  |  | *1.70E-02* |  |  |  | *1.39E-02* | *1.74E-02* |  |  | *2.92E-02* | *2.34E-02* |  |  |  |  |
|  | chr1:171179477 | T | *4.90E-02* | *0.28* |  |  |  |  |  |  | *4.90E-02* |  |  |  |  |  |  |  |  |  |  |  |  |  |  |  |
|  | chr1:171179779 | G | *5.08E-03* | *0.54* | *3.48E-02* |  |  | *2.84E-02* | *3.27E-02* |  |  | *2.47E-02* | *3.25E-02* |  |  | *2.48E-02* | *2.30E-02* | *2.73E-02* |  |  | *5.08E-03* | *1.54E-02* |  |  |  |  |
|  | chr1:171180071 | G | *5.08E-03* | *0.54* | *3.48E-02* |  |  | *2.84E-02* | *3.27E-02* |  |  | *2.47E-02* | *3.25E-02* |  |  | *2.48E-02* | *2.30E-02* | *2.89E-02* |  |  | *5.08E-03* | *1.54E-02* |  |  |  |  |
|  | chr1:171180201 | C | *5.08E-03* | *0.54* | *3.48E-02* |  |  | *2.84E-02* | *3.27E-02* |  |  | *2.47E-02* | *3.25E-02* |  |  | *2.48E-02* | *2.30E-02* | *2.77E-02* |  |  | *5.08E-03* | *1.54E-02* |  |  |  |  |
