# Supplementary material for: An ancestral human genetic variant linked to an ancient disease: A novel association of FMO2 polymorphisms with tuberculosis (TB) in Ethiopian populations provides new insight into the differential ethno-geographic distribution of FMO2*1
Source: PLoS One. 2017 Oct 5;12(10):e0184931. doi: 10.1371/journal.pone.0184931 (PMC5628799; doi:10.1371/journal.pone.0184931)
Supplement: S4 Table — (DOCX) [file pone.0184931.s008.docx]

S Table 4. Association test results in Test-model 2

| Test-model 2: Active TB vs. No LTBI | | | | | | | | | | | | | | | | | | | | | | | | | | |
| --- | --- | --- | --- | --- | --- | --- | --- | --- | --- | --- | --- | --- | --- | --- | --- | --- | --- | --- | --- | --- | --- | --- | --- | --- | --- | --- |
| Gene | SNP | Minor allele (A1) | Best p | OR | Fisher | | | | Pearson | | | | Logistic reg. | | | | Covariate | | | | | Stratified tests (CMH) | | | | |
|  |  |  |  |  | Combined | Merhabete | Adigrat | Arbaminch | Combined | Merhabete | Adigrat | Arbaminch | Combined | Merhabete | Adigrat | Arbaminch | Sex | Age | Mer-Adi | Mer-Arb | Adi-Arb | EGC | IBS | IBS-Mer | IBS-Adi | IBS-Arb |
|  | chr1:171165749 | T | 6.58E-05 | 15.9 | 6.58E-05 |  |  |  | 3.13E-04 |  |  |  | 4.87E-03 |  |  |  | 4.84E-03 | 4.15E-03 | 4.08E-02 |  | 1.57E-02 | 7.37E-04 | 2.38E-04 |  |  |  |
|  | chr1:171181877 | A | 3.19E-06 | 8.7 | 3.19E-06 |  | 2.96E-02 | 4.31E-03 | 2.20E-05 |  | 2.70E-02 | 5.88E-03 | 1.12E-04 |  | 4.29E-02 | 1.30E-02 | 1.17E-04 | 7.75E-05 | 1.09E-02 | 2.63E-03 | 1.26E-03 | 9.94E-05 | 3.01E-05 |  | 3.28E-02 |  |
|  | chr1:171174762 | C | 1.42E-02 | 0.15 | 1.90E-02 |  |  |  | 1.42E-02 |  |  |  | 1.48E-02 |  |  |  | 1.57E-02 | 1.73E-02 |  |  | 3.37E-02 |  |  |  |  |  |
|  | chr1:171178090 | C | 2.57E-02 | 0.50 |  |  |  |  | 4.06E-02 |  |  |  | 4.28E-02 |  |  |  | 4.11E-02 | 2.57E-02 |  |  |  |  |  |  |  |  |
|  | chr1:171179025 | C | 2.57E-02 | 0.50 |  |  |  |  | 4.06E-02 |  |  |  | 4.28E-02 |  |  |  | 4.11E-02 | 2.57E-02 |  |  |  |  |  |  |  |  |
|  | chr1:171179477 | T | 4.04E-02 | 0.57 |  |  |  |  |  |  |  |  |  |  |  |  |  |  | 4.04E-02 |  |  |  |  |  |  |  |
|  | chr1:171180201 | C | 3.72E-02 | 0.55 |  |  |  |  |  |  |  |  |  |  |  |  |  |  |  | 3.72E-02 |  |  |  |  |  |  |
