# Supplementary material for: An ancestral human genetic variant linked to an ancient disease: A novel association of FMO2 polymorphisms with tuberculosis (TB) in Ethiopian populations provides new insight into the differential ethno-geographic distribution of FMO2*1
Source: PLoS One. 2017 Oct 5;12(10):e0184931. doi: 10.1371/journal.pone.0184931 (PMC5628799; doi:10.1371/journal.pone.0184931)
Supplement: S5 Table — (DOCX) [file pone.0184931.s009.docx]

S Table 5. Association test results in Test-model 3

| Test-model 3: Active TB vs. LTBI | | | | | | | | | | | | | | | | | | | | | | | | | | | | | | |
| --- | --- | --- | --- | --- | --- | --- | --- | --- | --- | --- | --- | --- | --- | --- | --- | --- | --- | --- | --- | --- | --- | --- | --- | --- | --- | --- | --- | --- | --- | --- |
| Gene | SNP | Minor allele (A1) | Best p | OR | Fisher | | | | Pearson | | | | | Logistic reg. | | | | | Covariate | | | | | | Stratified tests (CMH) | | | | | |
|  |  |  |  |  | Combined | Merhabete | Adigrat | Arbaminch | | Combined | Merhabete | Adigrat | Arbaminch | | Combined | Merhabete | Adigrat | Arbaminch | | Sex | Age | Mer-Adi | Mer-Arb | Adi-Arb | | EGC | IBS | IBS-Mer | IBS-Adi | IBS-Arb |
| FMO2 | chr1:171165749 | T | 7.44E-04 | 5.7 | 7.44E-04 |  |  |  | | 1.44E-03 |  |  |  | | 2.86E-03 |  |  |  | | 2.84E-03 | 2.87E-03 |  |  | 8.51E-03 | | 1.68E-03 | 1.65E-03 |  |  |  |
|  | chr1:171179939 | G | 2.23E-02 | 1.7 |  |  |  |  | |  |  |  |  | |  |  |  |  | |  |  |  |  | 2.23E-02 | |  |  |  |  |  |
|  | chr1:171180021 | G | 2.23E-02 | 1.7 |  |  |  |  | |  |  |  |  | |  |  |  |  | |  |  |  |  | 2.23E-02 | |  |  |  |  |  |
|  | chr1:171181877 | A | 4.54E-04 | 5.9 | 5.92E-04 |  |  |  | | 9.32E-04 |  |  |  | | 6.11E-04 |  |  |  | | 6.02E-04 | 6.18E-04 |  | 6.52E-04 | 4.54E-04 | | 1.12E-03 | 1.40E-03 |  |  |  |
|  | chr1:171154303 | C | 4.00E-02 | 0.17 |  |  |  |  | |  |  | 4.00E-02 |  | |  |  | 4.93E-02 |  | |  |  |  |  |  | |  |  |  | 4.20E-02 |  |
|  | chr1:171168545 | C | 2.10E-02 | 0.21 |  |  | 3.63E-02 |  | |  |  | 2.23E-02 |  | |  |  | 2.50E-02 |  | |  | 2.50E-02 |  |  |  | |  |  |  | 2.10E-02 |  |
|  | chr1:171179779 | G | 2.40E-02 | 0.55 |  |  |  | 4.60E-02 | |  |  |  | 4.03E-02 | |  |  |  | 3.96E-02 | | 4.23E-02 |  |  |  | 2.40E-02 | | 4.84E-02 |  |  |  |  |
|  | chr1:171180071 | G | 2.40E-02 | 0.55 |  |  |  | 4.60E-02 | |  |  |  | 4.03E-02 | |  |  |  | 3.96E-02 | | 4.23E-02 |  |  |  | 2.40E-02 | | 4.84E-02 |  |  |  |  |
|  | chr1:171180201 | C | 3.83E-02 | 0.58 |  |  |  |  | |  |  |  |  | |  |  |  |  | |  |  |  |  | 3.83E-02 | |  |  |  |  |  |
