# Supplementary material for: An ancestral human genetic variant linked to an ancient disease: A novel association of FMO2 polymorphisms with tuberculosis (TB) in Ethiopian populations provides new insight into the differential ethno-geographic distribution of FMO2*1
Source: PLoS One. 2017 Oct 5;12(10):e0184931. doi: 10.1371/journal.pone.0184931 (PMC5628799; doi:10.1371/journal.pone.0184931)
Supplement: S6 Table — (DOCX) [file pone.0184931.s010.docx]

S Table 6. Association test results in Test-model 4

| Test-model 4: LTBI vs. No LTBI | | | | | | | | | | | | | | | | | | | | | | | | | | |
| --- | --- | --- | --- | --- | --- | --- | --- | --- | --- | --- | --- | --- | --- | --- | --- | --- | --- | --- | --- | --- | --- | --- | --- | --- | --- | --- |
| Gene | SNP | Minor allele (A1) | Best p | OR | Fisher | | | | Pearson | | | | Logistic reg. | | | | Covariate | | | | | Stratified tests (CMH) | | | | |
|  |  |  |  |  | Combined | Merhabete | Adigrat | Arbaminch | Combined | Merhabete | Adigrat | Arbaminch | Combined | Merhabete | Adigrat | Arbaminch | Sex | Age | Mer-Adi | Mer-Arb | Adi-Arb | EGC | IBS | IBS-Mer | IBS-Adi | IBS-Arb |
|  | chr1:171168545 | C | 2.21E-02 | 8.5 |  |  | 2.84E-02 |  |  |  | 2.21E-02 |  |  |  | 3.76E-02 |  |  |  |  |  |  |  |  |  | 2.88E-02 |  |
|  | chr1:171181877 | A | 4.13E-02 | 5.4 |  |  |  |  |  |  |  |  |  |  |  |  |  |  | 4.13E-02 |  |  |  |  |  |  |  |
|  | chr1:171179287 | T | 2.55E-02 | 0.04 |  | 3.21E-02 |  |  |  | 2.55E-02 |  |  |  |  |  |  |  |  |  |  |  |  |  | 2.83E-02 |  |  |
|  | chr1:171179477 | T | 4.54E-02 | 0.12 |  | 4.54E-02 |  |  |  |  |  |  |  |  |  |  |  |  |  |  |  |  |  |  |  |  |
|  | chr1:171179670 | G | 4.23E-02 | 0.04 |  | 4.23E-02 |  |  |  |  |  |  |  |  |  |  |  |  |  |  |  |  |  | 4.91E-02 |  |  |
|  | chr1:171179939 | G | 4.40E-02 | 0.35 |  |  |  |  |  |  |  |  |  |  |  |  |  |  |  |  |  |  |  |  |  | 4.40E-02 |
|  | chr1:171180021 | G | 4.40E-02 | 0.35 |  |  |  |  |  |  |  |  |  |  |  |  |  |  |  |  |  |  |  |  |  | 4.40E-02 |
