# Supplementary material for: An ancestral human genetic variant linked to an ancient disease: A novel association of FMO2 polymorphisms with tuberculosis (TB) in Ethiopian populations provides new insight into the differential ethno-geographic distribution of FMO2*1
Source: PLoS One. 2017 Oct 5;12(10):e0184931. doi: 10.1371/journal.pone.0184931 (PMC5628799; doi:10.1371/journal.pone.0184931)
Supplement: S7 Table — (DOCX) [file pone.0184931.s011.docx]

S Table 7. Summary of population-specific SNP-TB phenotype association test results

| **Best p-values per ethnicity (EGC)** | | | | | |
| --- | --- | --- | --- | --- | --- |
| **Gene** | **SNPs** | **Merhabete** | **Adigrat** | **Arbaminch** | **Combined population** |
| FMO2 | chr1:171154303 |  | 4.00E-02 |  |  |
|  | chr1:171165749 |  |  | 1.90E-04 | 3.32E-06 |
|  | chr1:171168469 |  |  |  | 4.72E-02 |
|  | chr1:171168545 |  | 2.10E-02 |  | 2.50E-02 |
|  | chr1:171173242 |  |  |  | 1.72E-02 |
|  | chr1:171174312 |  |  |  | 3.51E-02 |
|  | chr1:171174691 |  |  |  | 1.88E-02 |
|  | chr1:171174762 |  |  |  | 1.42E-02 |
|  | chr1:171174821 |  |  |  | 1.88E-02 |
|  | chr1:171176879 |  |  |  | 1.88E-02 |
|  | chr1:171177858 |  |  |  | 2.98E-02 |
|  | chr1:171178090 |  |  |  | 1.18E-02 |
|  | chr1:171178490 |  |  |  | 3.51E-02 |
|  | chr1:171179025 |  |  |  | 1.18E-02 |
|  | chr1:171179287 | 2.55E-02 |  |  |  |
|  | chr1:171179477 | 4.54E-02 | 4.90E-02 |  | 4.04E-02 |
|  | chr1:171179670 | 4.23E-02 |  |  |  |
|  | chr1:171179779 |  |  | 2.47E-02 | 5.08E-03 |
|  | chr1:171179939 |  | 2.45E-02 | 4.40E-02 | 2.23E-02 |
|  | chr1:171180021 |  | 2.45E-02 | 4.40E-02 | 2.23E-02 |
|  | chr1:171180071 |  |  | 2.47E-02 | 5.08E-03 |
|  | chr1:171180201 |  |  | 2.47E-02 | 5.08E-03 |
|  | chr1:171181150 |  |  |  | 4.72E-02 |
|  | chr1:171181877 |  | 2.70E-02 | 2.12E-06 | 3.15E-07 |
