# Supplementary material for: An ancestral human genetic variant linked to an ancient disease: A novel association of FMO2 polymorphisms with tuberculosis (TB) in Ethiopian populations provides new insight into the differential ethno-geographic distribution of FMO2*1
Source: PLoS One. 2017 Oct 5;12(10):e0184931. doi: 10.1371/journal.pone.0184931 (PMC5628799; doi:10.1371/journal.pone.0184931)
Supplement: S8 Table — (DOCX) [file pone.0184931.s012.docx]

S Table 8. Allele frequencies of TB phenotype-associated FMO2 SNPs among EGCs

| Minor allele frequency distribution of phenotype associated SNPs in "Active TB vs. No Active TB" dataset | | | | | | | | | | | | |
| --- | --- | --- | --- | --- | --- | --- | --- | --- | --- | --- | --- | --- |
| Gene | CHR | SNP | A1 | A2 | Combined Population | | Merhabete | | Adigrat | | Arbaminch | |
|  |  |  |  |  | F_A1-A | F_A1_U | F_A1-A | F_A1_U | F_A1-A | F_A1_U | F_A1-A | F_A1_U |
| FMO2 | 1 | chr1:171154303 | C | A | 0.183 | 0.2158 | 0.1613 | 0.2179 | 0.1579 | 0.2447 | 0.2024 | 0.1887 |
|  | 1 | chr1:171165749 | T | G | 0.1111 | 0.01799 | 0.08065 | 0 | 0.09211 | 0.04255 | 0.131 | 0.009434 |
|  | 1 | chr1:171168469 | A | C | 0.03595 | 0.01079 | 0 | 0.01282 | 0.01316 | 0 | 0.05952 | 0.01887 |
|  | 1 | chr1:171168545 | C | T | 0.03595 | 0.04676 | 0 | 0.03846 | 0.03947 | 0.08511 | 0.04762 | 0.01887 |
|  | 1 | chr1:171173242 | C | T | 0.1405 | 0.2086 | 0.1129 | 0.141 | 0.1316 | 0.2234 | 0.1548 | 0.2453 |
|  | 1 | chr1:171174312 | A | T | 0.1438 | 0.08993 | 0.1935 | 0.1282 | 0.1316 | 0.06383 | 0.131 | 0.08491 |
|  | 1 | chr1:171174691 | A | G | 0.1373 | 0.205 | 0.1129 | 0.141 | 0.1316 | 0.2234 | 0.1488 | 0.2358 |
|  | 1 | chr1:171174762 | C | G | 0.03595 | 0.08273 | 0.06452 | 0.0641 | 0.06579 | 0.1489 | 0.0119 | 0.03774 |
|  | 1 | chr1:171174821 | A | G | 0.1373 | 0.205 | 0.1129 | 0.141 | 0.1316 | 0.2234 | 0.1488 | 0.2358 |
|  | 1 | chr1:171176879 | A | G | 0.1373 | 0.205 | 0.1129 | 0.141 | 0.1316 | 0.2234 | 0.1488 | 0.2358 |
|  | 1 | chr1:171177858 | T | G | 0.05882 | 0.1079 | 0.06452 | 0.0641 | 0.09211 | 0.1702 | 0.04167 | 0.08491 |
|  | 1 | chr1:171178090 | C | T | 0.1013 | 0.1727 | 0.1129 | 0.141 | 0.1316 | 0.2234 | 0.08333 | 0.1509 |
|  | 1 | chr1:171178490 | T | C | 0.1438 | 0.08993 | 0.1935 | 0.1282 | 0.1316 | 0.06383 | 0.131 | 0.08491 |
|  | 1 | chr1:171179025 | C | T | 0.1013 | 0.1727 | 0.1129 | 0.141 | 0.1316 | 0.2234 | 0.08333 | 0.1509 |
|  | 1 | chr1:171179287 | T | C | 0.1863 | 0.1835 | 0.1452 | 0.1667 | 0.1579 | 0.2128 | 0.2143 | 0.1698 |
|  | 1 | chr1:171179477 | T | C | 0.2876 | 0.3561 | 0.2581 | 0.3077 | 0.2895 | 0.4362 | 0.2976 | 0.3208 |
|  | 1 | chr1:171179670 | G | C | 0.1503 | 0.1547 | 0.1452 | 0.1538 | 0.1184 | 0.1489 | 0.1667 | 0.1604 |
|  | 1 | chr1:171179779 | G | A | 0.2908 | 0.3741 | 0.2742 | 0.2821 | 0.2632 | 0.3723 | 0.3095 | 0.4434 |
|  | 1 | chr1:171179939 | G | T | 0.5229 | 0.4496 | 0.4194 | 0.4487 | 0.5789 | 0.4149 | 0.4762 | 0.4057 |
|  | 1 | chr1:171180021 | G | T | 0.5229 | 0.4496 | 0.4194 | 0.4487 | 0.5789 | 0.4149 | 0.4762 | 0.4057 |
|  | 1 | chr1:171180071 | G | A | 0.2908 | 0.3741 | 0.2742 | 0.2821 | 0.2632 | 0.3723 | 0.3095 | 0.4434 |
|  | 1 | chr1:171180201 | C | T | 0.2908 | 0.3741 | 0.2742 | 0.2821 | 0.2632 | 0.3723 | 0.3095 | 0.4434 |
|  | 1 | chr1:171181150 | A | G | 0.03595 | 0.01079 | 0 | 0.01282 | 0.01316 | 0 | 0.05952 | 0.01887 |
|  | 1 | chr1:171181877 | A | C | 0.1732 | 0.04317 | 0.1129 | 0.05128 | 0.1447 | 0.06383 | 0.2083 | 0.01887 |

F_A1_A=Freq. of A1 allele in affecteds/cases; F_A1_U=Freq. of A1 in unaffecteds/controls
