# Supplementary material for: An ancestral human genetic variant linked to an ancient disease: A novel association of FMO2 polymorphisms with tuberculosis (TB) in Ethiopian populations provides new insight into the differential ethno-geographic distribution of FMO2*1
Source: PLoS One. 2017 Oct 5;12(10):e0184931. doi: 10.1371/journal.pone.0184931 (PMC5628799; doi:10.1371/journal.pone.0184931)
Supplement: S9 Table — (DOCX) [file pone.0184931.s013.docx]

S Table 9: Breslow-Day test results for hetrogeneous associations

| Heterogenous association between ethno-geographic categories | | | | | | | | | | | | |
| --- | --- | --- | --- | --- | --- | --- | --- | --- | --- | --- | --- | --- |
| Test-model | Gene | SNP | BP | A1 | MAF | A2 | CHISQ | P | OR | U95 | CHISQ_BD | P_BD |
| Active TB vs. LTBI | FMO2 | chr1:171165749 | 171165749 | T | 0.08296 | G | 9.866 | 0.001684 | 5.891 | 20.09 | 6.85E+00 | 0.03248 |
|  |  | chr1:171181877 | 171181877 | A | 0.1368 | C | 10.62 | 0.001117 | 3.448 | 7.527 | 9.939 | 0.006948 |
