# Supplementary material for: An ancestral human genetic variant linked to an ancient disease: A novel association of FMO2 polymorphisms with tuberculosis (TB) in Ethiopian populations provides new insight into the differential ethno-geographic distribution of FMO2*1
Source: PLoS One. 2017 Oct 5;12(10):e0184931. doi: 10.1371/journal.pone.0184931 (PMC5628799; doi:10.1371/journal.pone.0184931)
Supplement: S10 Table — (DOCX) [file pone.0184931.s014.docx]

S Table 10. Test results for allele frequency differences

| Phenotype-associated SNPs showing significant allele frequency difference in "Active TB vs. No Active TB" dataset | | | | | | | | |
| --- | --- | --- | --- | --- | --- | --- | --- | --- |
| Gene | Compared EGC  (EGC1 vs. EGC2) | SNP | A1 | F_EGC1 | F_EGC2 | A2 | CHISQ | P |
| FMO2 | Adigrat vs. Arbaminch | chr1:171168469 | A | 0.0438 | 0.005882 | C | 5.306 | 0.02126 |
|  | Merhabete vs. Arbaminch |  |  | 0.0438 | 0.007143 | C | 4.093 | 0.04307 |
|  | Adigrat vs. Arbaminch* | chr1:171174762 | C | 0.0219 | 0.1118 | G | 15.95 | 6.52E-05* |
|  | Merhabete vs. Arbaminch |  |  | 0.0219 | 0.06429 | G | 4.768 | 0.029 |
|  | Adigrat vs. Arbaminch | chr1:171177858 | T | 0.05839 | 0.1353 | G | 7.743 | 0.00539 |
|  | Merhabete vs. Adigrat |  |  | 0.1369 | 0.06429 | G | 4.325 | 0.03755 |
|  | Adigrat vs. Arbaminch | chr1:171178090 | C | 0.1095 | 0.1824 | T | 4.7 | 0.03017 |
|  | Adigrat vs. Arbaminch | chr1:171179025 | C | 0.1095 | 0.1824 | T | 4.7 | 0.03017 |
|  | Merhabete vs. Arbaminch | chr1:171179939 | G | 0.4489 | 0.5643 | T | 4.937 | 0.02629 |
|  | Merhabete vs. Arbaminch | chr1:171180021 | G | 0.4489 | 0.5643 | T | 4.937 | 0.02629 |
|  | Adigrat vs. Arbaminch | chr1:171181150 | A | 0.0438 | 0.005882 | G | 5.306 | 0.02126 |
|  | Merhabete vs. Arbaminch |  |  | 0.0438 | 0.007143 | G | 4.093 | 0.04307 |

*=Significant after Bonf. correction for multiple testing
