# Supplementary material for: An ancestral human genetic variant linked to an ancient disease: A novel association of FMO2 polymorphisms with tuberculosis (TB) in Ethiopian populations provides new insight into the differential ethno-geographic distribution of FMO2*1
Source: PLoS One. 2017 Oct 5;12(10):e0184931. doi: 10.1371/journal.pone.0184931 (PMC5628799; doi:10.1371/journal.pone.0184931)
Supplement: S11 Table — (DOCX) [file pone.0184931.s015.docx]

S Table 11: Functional consequences of mutations risk (Annotation was done using Homosapiens/UCSC/hg19)

| GENE | chr#:base_position | rs_ID | A1  (Minor allele) | A2  (Major allele) | PREDICTED FUNCTION (UCSC) |
| --- | --- | --- | --- | --- | --- |
| FMO2 | chr1:171154303 | rs28369794 | C | A | UPSTREM GENE VARIANT |
|  | chr1:171165749 | NOVEL VARIANT | T | G |  |
|  | chr1:171168469 | rs112884205 | A | C | INTRON VARIANT |
|  | chr1:171168545 | rs2307492 | C | T | INTRON VARIANT, MISSENSE VARIANT |
|  | chr1:171173242 | rs7517460 | C | T | INTRON VARIANT |
|  | chr1:171174312 | rs16864177 | A | T | INTRON VARIANT (IN STROG LD WITH A MISSENSE VARIANT) |
|  | chr1:171174691 | rs7536646 | A | G | SYNONYMOUS VARIANT |
|  | chr1:171174762 | rs28369899 | C | G | MISSENSE VARIANT |
|  | chr1:171174821 | rs7536745 | A | G | INTRON VARIANT |
|  | chr1:171176879 | rs6671692 | A | G | SYNONYMOUS VARIANT, INTRON VARIANT |
|  | chr1:171177858 | rs28369911 | T | G | INTRON VARIANT |
|  | chr1:171178090 | rs6661174 | C | T | STOP GAINED, 3' UTR VARIANT (NONSENSE) |
|  | chr1:171178490 | rs28369914 | T | C | DOWNSTREAM GENE VARIANT, 3' UTR VARIANT |
|  | chr1:171179025 | rs6664553 | C | T | 3' UTR VARIANT |
|  | chr1:171179287 | rs7512785 | T | C | 3' UTR VARIANT |
|  | chr1:171179477 | rs7515157 | T | C | 3' UTR VARIANT |
|  | chr1:171179670 | rs28369918 | G | C | 3' UTR VARIANT |
|  | chr1:171179779 | rs73032526 | G | A | 3' UTR VARIANT |
|  | chr1:171179939 | rs3174837 | T | G | 3' UTR VARIANT, SPLICE REGION VARIANT, INTRON VARIANT |
|  | chr1:171180021 | rs6425286 | T | G | 3' UTR VARIANT |
|  | chr1:171180071 | rs6673781 | G | A | 3' UTR VARIANT |
|  | chr1:171180201 | rs6668231 | C | T | 3' UTR VARIANT (N STRONG LD WITH A MISSENSE VARIANT) |
|  | chr1:171181150 | rs113252377 | A | G | 3' UTR VARIANT |
|  | chr1:171181877 | NOVEL VARIANT | A | C |  |
