# Supplementary material for: An ancestral human genetic variant linked to an ancient disease: A novel association of FMO2 polymorphisms with tuberculosis (TB) in Ethiopian populations provides new insight into the differential ethno-geographic distribution of FMO2*1
Source: PLoS One. 2017 Oct 5;12(10):e0184931. doi: 10.1371/journal.pone.0184931 (PMC5628799; doi:10.1371/journal.pone.0184931)
Supplement: S13 Table — (DOCX) [file pone.0184931.s017.docx]

S Table 13: Sequenced regions of *FMO2*

| Genomic coordinates captured for sequencing using Homosapiens/UCSC/hg19 | | | | | | | |
| --- | --- | --- | --- | --- | --- | --- | --- |
| Target | Chromosome | Start Coordinate | Stop Coordinate | Length | Padding Per Exon | Coverage | Score |
| FMO2 + FMO2 | 1 | 171154363 | 171155009 | 647 | 0 | 100 | 78 |
| FMO2_Exon_2288527 | 1 | 171162449 | 171162687 | 239 | 25 | 100 | 96 |
| FMO2_Exon_2288528 | 1 | 171165763 | 171165975 | 213 | 25 | 100 | 96 |
| FMO2_Exon_2288779 | 1 | 171168460 | 171168652 | 193 | 25 | 100 | 96 |
| FMO2_Exon_2288529 | 1 | 171172979 | 171173228 | 250 | 25 | 100 | 96 |
| FMO2_Exon_2289044 | 1 | 171174393 | 171174798 | 406 | 25 | 100 | 96 |
| FMO2_Exon_2288618 | 1 | 171176832 | 171176954 | 123 | 25 | 100 | 96 |
| FMO2 + FMO2 | 1 | 171177908 | 171181847 | 3940 | 0 | 100 | 75 |
