# Supplementary material for: An ancestral human genetic variant linked to an ancient disease: A novel association of FMO2 polymorphisms with tuberculosis (TB) in Ethiopian populations provides new insight into the differential ethno-geographic distribution of FMO2*1
Source: PLoS One. 2017 Oct 5;12(10):e0184931. doi: 10.1371/journal.pone.0184931 (PMC5628799; doi:10.1371/journal.pone.0184931)
Supplement: Supplementary file 15 [file pone.0184931.s002.docx]

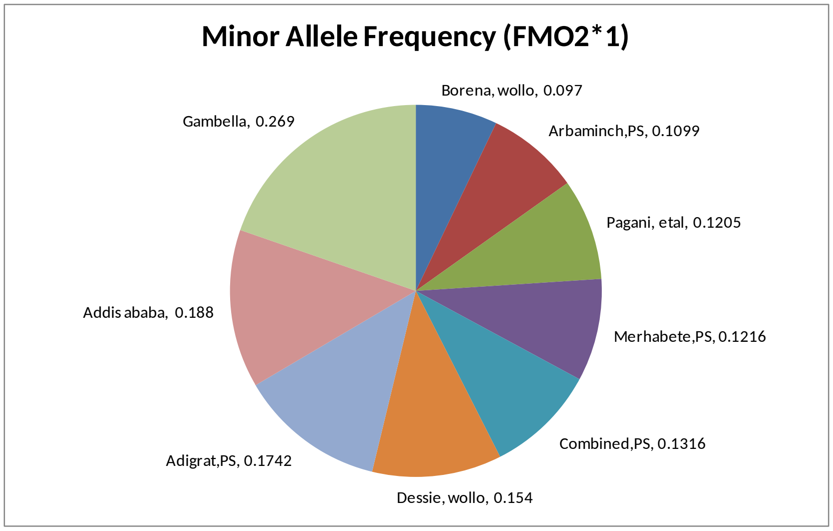
Minor Allele Frequency (FMO2*1)
Borena, wollo, 0.097
Arbaminch,PS, 0.1099
Pagani, etal, 0.1205
Merhabete,PS, 0.1216
Combined,PS, 0.1316
Dessie, wollo, 0.154
Adigrat,PS, 0.1742
Addis ababa, 0.188
Gambella, 0.269
