# Supplementary material for: An ancestral human genetic variant linked to an ancient disease: A novel association of FMO2 polymorphisms with tuberculosis (TB) in Ethiopian populations provides new insight into the differential ethno-geographic distribution of FMO2*1
Source: PLoS One. 2017 Oct 5;12(10):e0184931. doi: 10.1371/journal.pone.0184931 (PMC5628799; doi:10.1371/journal.pone.0184931)
Supplement: Supplementary file 16 [file pone.0184931.s003.docx]

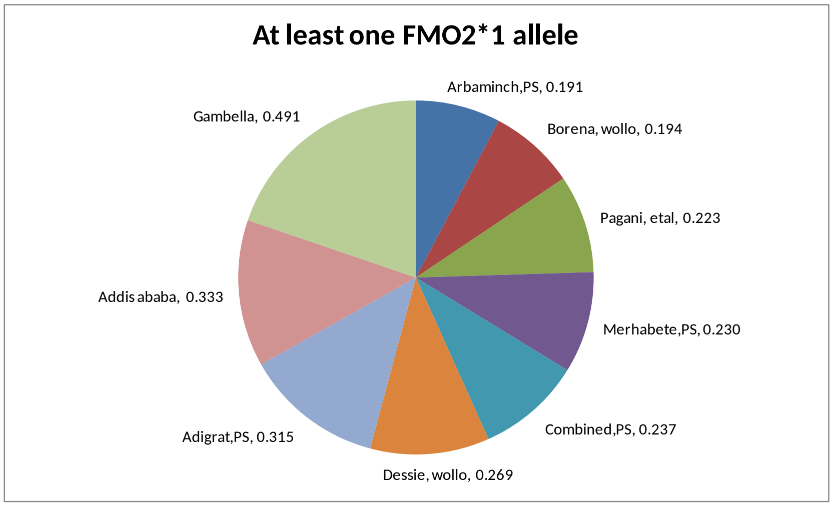
At least one FMO2*1 allele
Arbaminch,PS, 0.191
Borena, wollo, 0.194
Pagani, etal, 0.223
Merhabete,PS, 0.230
Combined,PS, 0.237
Dessie, wollo, 0.269
Adigrat,PS, 0.315
Addis ababa, 0.333
Gambella, 0.491
