# Supplementary material for: An ancestral human genetic variant linked to an ancient disease: A novel association of FMO2 polymorphisms with tuberculosis (TB) in Ethiopian populations provides new insight into the differential ethno-geographic distribution of FMO2*1
Source: PLoS One. 2017 Oct 5;12(10):e0184931. doi: 10.1371/journal.pone.0184931 (PMC5628799; doi:10.1371/journal.pone.0184931)
Supplement: Supplementary file 17 [file pone.0184931.s004.docx]

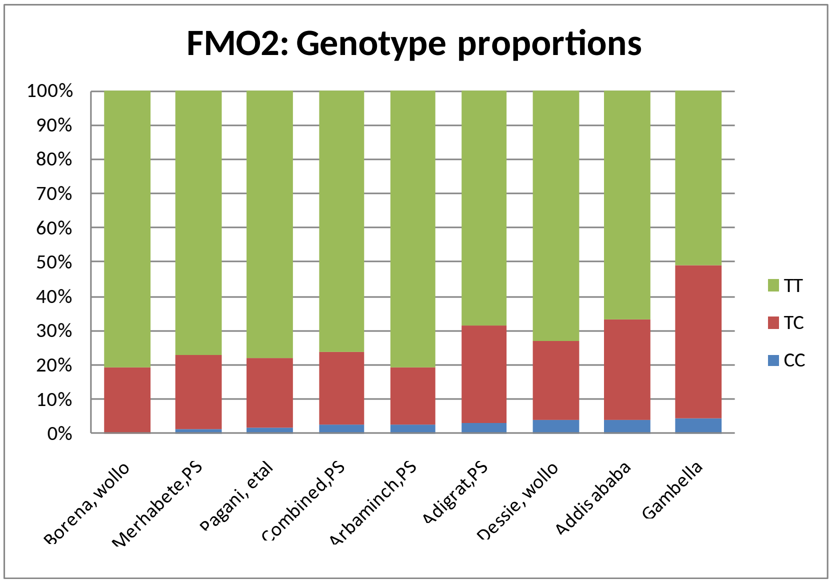
FMO2: Genotype proportions
100%
90%
80%
70%
60%
50%
40%
30%
20%
10%
0%
Borena, wollo
Merhabete,PS
Pagani, etal
Combined,PS
Arbaminch,PS
Adigrat,PS
Dessie, wollo
Addis ababa
Gambella
TT
TC
CC
